# T cell responses to nonstructural proteins promote cross-serotype immunity to foot-and-mouth disease virus

**DOI:** 10.1128/mbio.03586-25

**Published:** 2026-04-06

**Authors:** Suyu Mu, Shaobin Shang, Hu Dong, Shuang Wang, Lingbo Chen, Zhihui Zhang, Manyuan Bai, Zhidong Teng, Yaozhong Ding, Yun Zhang, Huichen Guo, Shiqi Sun

**Affiliations:** 1 State Key Laboratory for Animal Disease Control and Prevention, College of Veterinary Medicine, Lanzhou University, Lanzhou Veterinary Research Institute, Chinese Academy of Agricultural Sciences 111658 , Lanzhou, China; 2 College of Veterinary Medicine, Institute of Comparative Medicine, Yangzhou University, Yangzhou, China; Huazhong Agricultural University, Wuhan, Hubei, China

**Keywords:** foot-and-mouth disease virus, T-cell epitope, cellular immune response, CD40L, cross-protection

## Abstract

T-cell responses play a pivotal role in protection against foot-and-mouth disease virus (FMDV), by enhancing neutralizing antibody (NAb) titers and shortening the duration of infection. To further elucidate the protective immune mechanisms mediated by T cells and develop novel candidate vaccines with cross-protective potential, multiple CD4^+^ and CD8^+^ T-cell epitopes were identified targeting FMDV nonstructural proteins 2B and 2C in the infected pigs. Given its critical role in dendritic cells targeting and enhancing antigen presentation, CD40L was fused with T-/B-cell epitopes to construct a dendritic cell-targeting broad-spectrum T-cell epitope trimer antigen Trimer-CD40L-TB. When compared to the control antigen Trimer-B (only B-cell epitopes), both Trimer-CD40L-TB and CD40L-free Trimer-TB effectively stimulated multifunctional CD4^+^ and CD8^+^ T-cell responses and significantly enhanced humoral immunity induced by the FMDV GH-Loop peptide, which provided more effective broad-spectrum protection against FMDV serotypes A and O. The underlying mechanisms involved Th1 response activation, memory T-cell differentiation, germinal center formation, and high-level NAb production. The synergy between epitope-specific T cells and humoral immunity is key to protective immune responses. This study presents a novel strategy for the development of broad-spectrum vaccines against FMDV.

**IMPORTANCE:**

CD4^+^ and CD8^+^ T-cell epitopes of the nonstructural proteins 2B and 2C of foot-and-mouth disease virus (FMDV) were systematically characterized using FMDV-infected swine combined with intracellular cytokine staining and IFN-γ ELISpot assays. The highly conserved epitopes 2C 157–174 and 2C 109–126, identified across seven FMDV serotypes, were recognized by both swine and cattle T cells. This discovery will aid in the development of universal FMDV vaccines capable of eliciting cross-serotype and cross-species immunity. Unlike conventional FMDV vaccines that rely on humoral responses, the trimeric vaccine Trimer-CD40L-TB, integrating CD40L-enhanced T cells, was engineered with conserved B-/T-cell epitopes. This design not only induced broad-spectrum Th1/cytotoxic T-cell responses but also improved germinal center formation and neutralizing antibody production, leading to cross-protection against serotypes O and A. This approach demonstrated that T-cell-mediated immunity is essential for durable and cross-serotype protection, thereby offering a blueprint for next-generation vaccines.

## INTRODUCTION

Foot-and-mouth disease (FMD) is an acute and highly contagious infectious disease of cloven-hoofed animals caused by the foot-and-mouth disease virus (FMDV), characterized by high fever, vesicular lesions of the mucosa, and a high mortality rate (1). The FMDV genome is a positive-sense single RNA strand of approximately 8,500 nucleotides, enclosed within an icosahedral capsid that encodes four structural proteins (VP1, VP2, VP3, and VP4) and seven nonstructural proteins (NSPs; 2A, 2B, 2C, 3A, 3B, 3C, and 3D) (2). FMDV includes seven distinct serotypes (O, A, C, SAT1, SAT2, SAT3, and Asia1) without cross-protection between each serotype (3). More than 140 outbreaks of FMD caused by FMDV serotypes O and A have occurred in China since 2010 (4). Although it is the primary control measure, type-specific inactivated vaccines fail to provide effective cross-protection. This is primarily due to the highly serotype-specific variations at key antigenic sites, such as the VP1 G-H loop epitopes 135–156 (5). Therefore, novel FMDV vaccines with broad-spectrum protective efficacy are urgently needed.

Multiple broad-spectrum vaccine candidates targeting the VP1 135–156 epitopes of the G-H loop across different FMDV serotypes only induce low neutralizing antibody (NAb) titers and partial protection in the absence of T-cell responses (3), highlighting the crucial role of T cells in the development of vaccines against FMDV. CD4^+^ T cells play a central role in anti-FMDV immunity by supporting B-cell antibody (Ab) production (including NAb titers and class switching) and enhancing CD8^+^ T-cell function (6, 7). Incorporating identified CD4^+^ T-cell epitopes of FMDV NSPs (e.g., 3A, 3C, 3D) into vaccine antigens can significantly boost immune responses against the VP1 G-H loop (8–11). CD8^+^ T cells (cytotoxic T lymphocytes, CTLs) contribute to the clearance of virus-infected cells (12). FMDV-induced CTL activation has been shown to reduce disease duration and viremia in cattle (13) and is associated with early protection in swine (14). Although CD8^+^ T-cell epitopes have been identified within FMDV structural proteins (e.g., VP1) (15), their potential roles in vaccination remain unclear, as highly efficient T-cell epitopes (particularly CTL epitopes) derived from FMDV NSPs remain poorly characterized.

The NSPs 2B and 2C are highly conserved among different FMDV strains (16) and exhibit significant immunogenic potential. Studies have demonstrated that the FMDV 2B protein enhances vaccine efficacy and induces cellular immunity (17), while the 2C protein contains novel antiviral sites (18). Notably, a DNA vaccine targeting the 2C protein was reported to elicit CTL responses in mice (19). These findings suggest that 2B and 2C proteins may harbor previously unidentified porcine CD4^+^ and CD8^+^ T-cell epitopes with critical functional importance. However, our current understanding of early T-cell responses in FMDV-infected swine remains inadequate (20). Consequently, conventional strategies for identification of T-cell epitopes of NSPs (e.g., screening during convalescent phases) may be suboptimal (21). Our preliminary studies revealed that all NSPs were detectable in peripheral blood cells as early as 7 days post-infection in naturally infected pigs (22), coinciding with robust CD4^+^ and CD8^+^ T-cell proliferation by day 14 (14), before gradually subsiding. This temporal pattern indicates that the early infection phase—rather than late convalescence (21)—is likely a critical window to identify NSP-specific T-cell epitopes, particularly CTL epitopes.

Beyond epitope identification, efficient antigen presentation is crucial for eliciting robust T-/B-cell responses and the establishment of immune memory. Activated dendritic cells (DCs), as professional antigen-presenting cells (APCs), play a pivotal role in initiating adaptive immunity (23, 24). Targeting the CD40 molecule on DC surfaces presents a highly promising strategy. The soluble CD40 ligand (sCD40L) trimer effectively binds to CD40 of DCs (and B cells), mimicking natural CD40L-CD40 interactions to deliver potent co-stimulatory signals (25). Fusion expressions of sCD40L with viral antigens (such as influenza HA, classical swine fever E2, porcine reproductive and respiratory syndrome virus GP3/GP5, and bovine alphaherpesvirus 1 gD) have been shown to enhance antigen-specific responses of type 1 and 2 helper T (Th1/2) cells, CTL activation, NAb titers, and protective efficacy in murine and porcine models (26–30). *In vitro* studies have also confirmed that sCD40L can effectively activate porcine DCs and promote the polarization of naïve T cells toward the Th1 phenotype (31). However, native sCD40L trimer has limited conformational stability. Research has demonstrated that incorporating trimerization motifs like the isoleucine zipper (IZ) can significantly improve the bioactivity of sCD40L (32, 33). For example, IZ-modified sCD40L combined with inactivated *Staphylococcus aureus* was reported to induce strong Th1 and CTL responses (33). However, this enhanced sCD40L trimer-based presentation strategy remains unexplored in swine FMDV vaccines.

Therefore, in this study, overlapping peptide technology was used to comprehensively screen pig-derived T-cell epitopes (especially CTL epitopes) in FMDV conserved proteins 2B and 2C, while fine-level identification and functional verification were assessed by flow cytometry (phenotype, proliferation) and swine leukocyte antigen (SLA) blocking experiments. In addition, a fusion antigen was constructed by in tandem expressing the trimerization motif dn5b, porcine sCD40L (residues 113–261), key T-cell epitopes, and/or FMDV cross B-cell epitope (VP1 residues 137–156) with the *E. coli* system (named Trimer-CD40L-TB). The results showed that Trimer-CD40L-TB induced an FMDV-specific Th1 response, CTL activation, memory T-cell formation, germinal center (GC) reaction, and high-titer cross-NAbs, which resulted in broad-spectrum protection in the immunized pigs against FMDV type O and A. The role of sCD40L and newly identified T-cell epitopes in enhancing these responses was further clarified. Our study systematically revealed the functions of conserved T-cell epitopes (especially CTL epitopes) of FMDV 2B and 2C proteins. More importantly, the enhanced sCD40L trimer-targeted presentation strategy was applied to the design of the FMDV vaccine for pigs, which provides an important theoretical basis and technical solutions for developing the next generation of FMDV vaccines with high cross-protection.

## RESULTS

### Kinetics of T-cell responses to FMDV nonstructural proteins 2B and 2C

**Fig 1:**
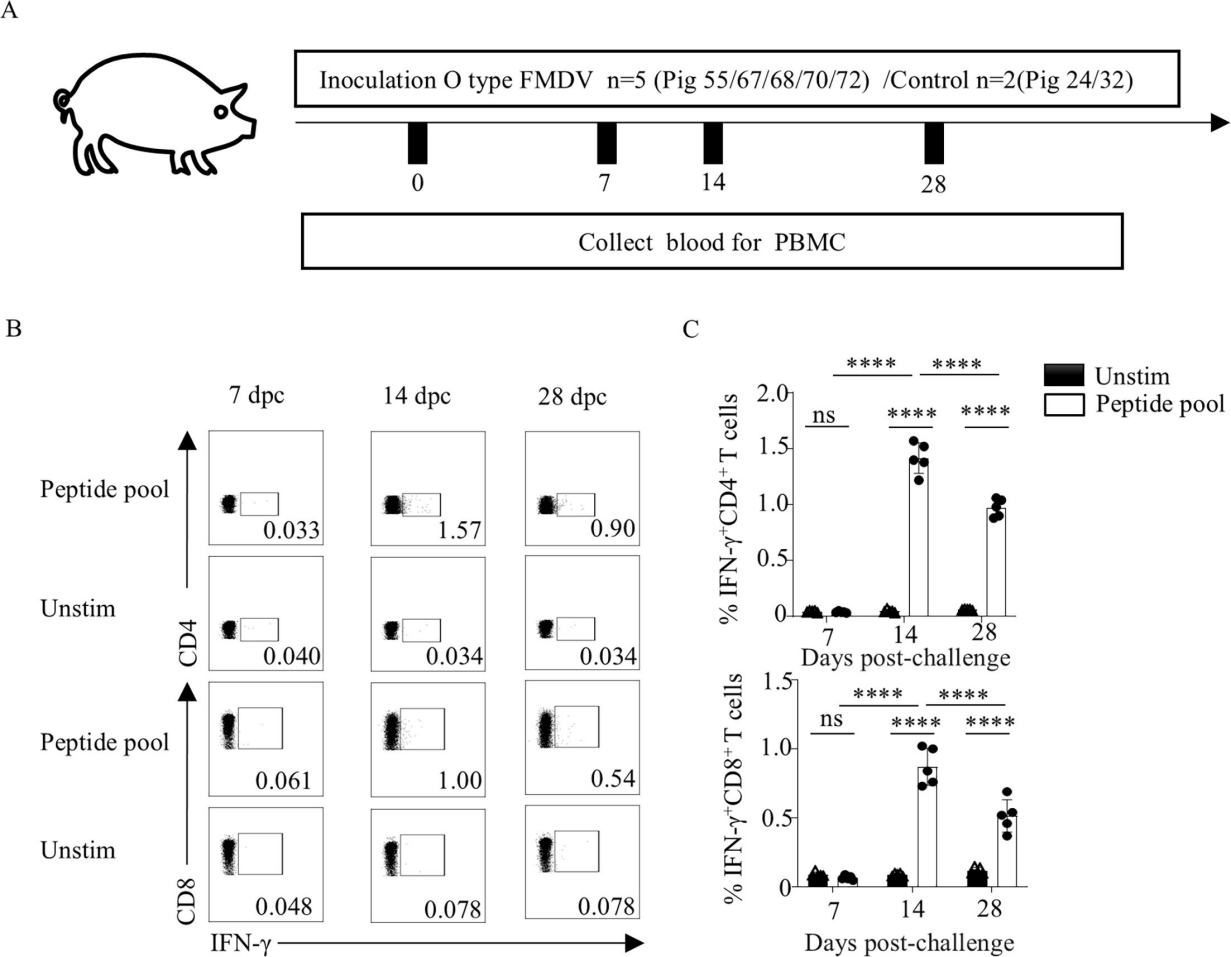
IFN-γ responses of PBMCs from FMDV-infected pigs to synthetic peptide pools. PBMCs were isolated from pigs infected with FMDV O/BY/2010, stimulated *in vitro* with peptide pools composed of 75 conserved peptide segments of FMDV O/BY/2010, and stained for intracellular cytokines to detect the IFN-γ reactivity of CD4^+^ and CD8^+^ T cells. (**A**) Schematic diagram showing the number of pigs infected with serotype O FMDV and sample collection. (**B and C**) Kinetics of IFN-γ responses in PBMCs from five infected pigs stimulated with FMDV peptide pools. Measurements were performed using PBMCs obtained at 0, 7, 14, and 28 dpc. Note: due to the absence of epitope-specific IFN-γ response detected at day 0 of infection, data at this time point are not displayed. (**B**) Representative dot plots; (**C**) percentage of peptide-specific IFN-γ-producing CD4^+^ and CD8^+^ T cells. Data are presented as the mean ± standard deviation (*n* = 5). The data were assessed by two-way ANOVA, ns: not significant. *P < 0.05, **P < 0.01, ***P < 0.001, ****P < 0.0001.

To characterize the kinetics of T-cell responses specific to FMDV NSP 2B and 2C (Fig. 1A), intracellular cytokine staining (ICS) was used to quantify the frequencies of IFN-γ-secreting CD4^+^ and CD8^+^ T cells in PBMCs from FMDV-infected pigs at 7, 14, and 28 dpc following stimulation with 2B and 2C peptide pools. Significantly elevated frequencies of IFN-γ^+^ CD4^+^ and CD8^+^ T cells were observed at 14 and 28 dpc compared to unstimulated controls. The responses peaked at 14 dpc, with significantly higher levels than those at 28 dpc (Fig. 1B and C). These findings indicate that FMDV NSP 2B and 2C peptide pools contain immunodominant epitopes capable of activating porcine CD4^+^ and CD8^+^ T cells.

### Identification of T-cell epitopes in FMDV nonstructural proteins 2B and 2C

Based on the observed strong T-cell response specific to 2B/2C peptide pool, 75 overlapping peptides (Table S1) covering the 2B and 2C proteins were synthesized and grouped into 19 peptide pools in a checkerboard (Table S2). To identify T-cell epitopes, PBMCs collected at 14 dpc were used for initial screening of effector T-cell responses, while PBMCs obtained at 28 dpc were employed to assess memory T-cell responses, in accordance with the response kinetics shown in Fig. 1.

**Fig 2:**
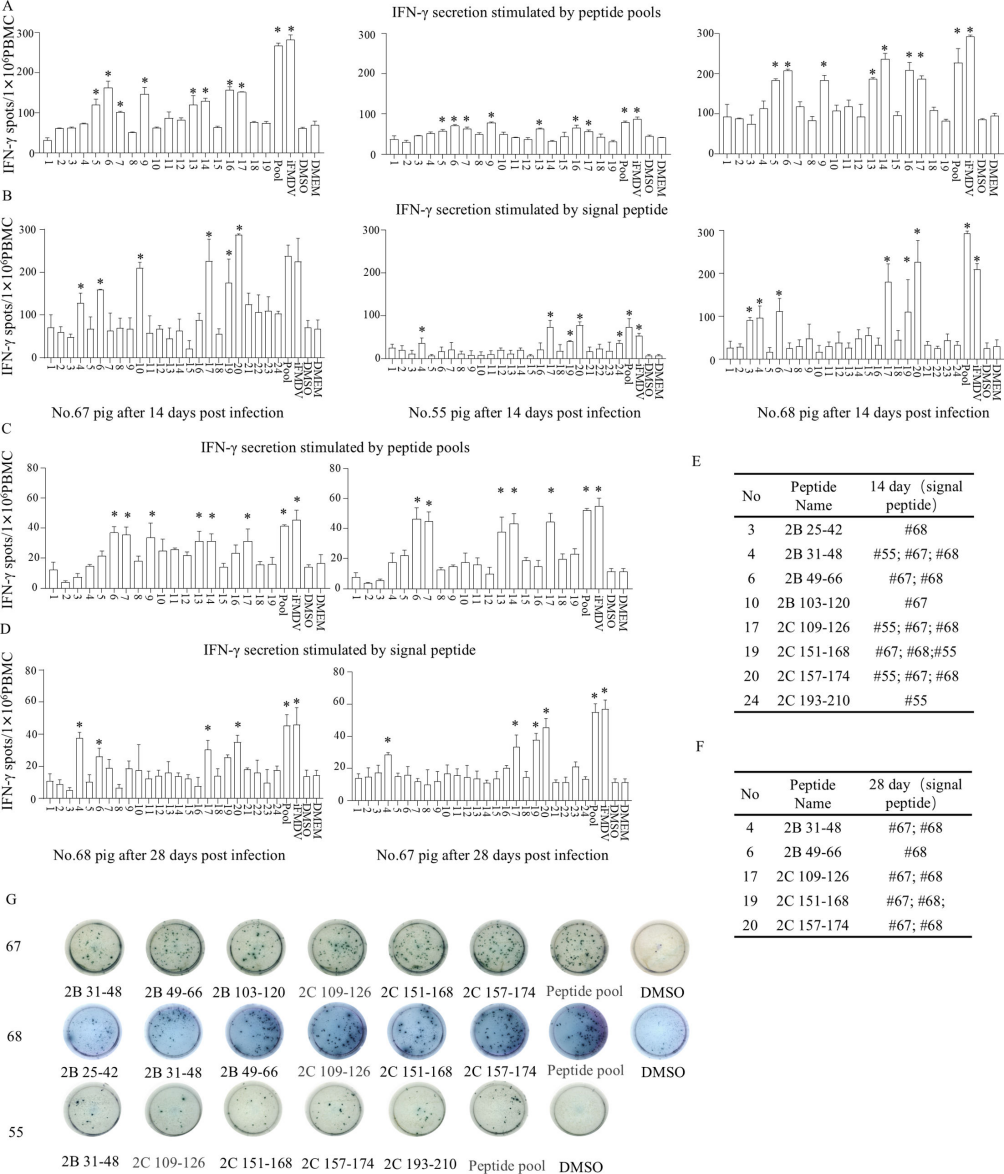
Screening of T-cell epitopes in FMDV NSP 2B and 2C by IFN-γ ELISpot. PBMCs were isolated from pigs #67, #55, and #68 at 14 or 28 days post-infection. Peptide pools or individual peptides were used to stimulate PBMCs for 36 h, followed by the ELISpot assay to detect IFN-γ secretion. (**A and C**) PBMCs from pigs #67, #55, and #68 at 14 and 28 days post-infection were stimulated with 19 peptide pools. A mixture of the 19 peptide pools and inactivated FMDV (iFMDV) served as positive controls; Dimethyl sulfoxide and DMEM served as negative controls. (**B and D**) Twenty-four individual peptides identified by cross-screening as potentially reactive were used to stimulate PBMCs from pigs #67, #55, and #68 at 14 or 28 days post-infection. A mixture of the 24 individual peptides and iFMDV served as positive controls; Dimethyl sulfoxide and DMEM served as negative controls, #55 pig in the 22 days post-infection was dead. (**E and F**) Statistics of individual peptides showing IFN-γ reactivity and recognition at 14 or 28 days post-infection. (**G**) Representative ELISpot images of reactive individual peptides, negative controls, and positive controls. Repeat three times for each sample (*n* = 3), and the data are presented as the mean ± standard deviation. The data were assessed by one-way ANOVA, ns: not significant, *P < 0.05, significantly different from the negative control.

IFN-γ ELISpot assays showed that peptide pools 5, 6, 7, 9, 13, 14, 16, and 17 significantly induced IFN-γ secretion (Fig. 2A; Table S2). Subsequent screening with individual peptides (Table S4) from these positive pools identified four peptides (2B 31–48, 2C 109–126, 2C 151–168, and 2C 157–174) that elicited strong IFN-γ responses upon stimulation of PBMCs at 14 dpc (Fig. 2B, E and G). When PBMCs at 28 dpc were stimulated with the corresponding peptide pool and key single peptides (Fig. 2C and D), peptides 2B 31–48, 2C 109–126, 2C 151–168, and 2C 157–174 were still capable of activating memory T cells to secrete IFN-γ. In contrast, PBMCs from the control (DMEM-treated) pigs showed no response to any of the 2B- and 2C-derived peptides (data not shown).

### Phenotypic analysis of peptide-specific T cells

**Fig 3:**
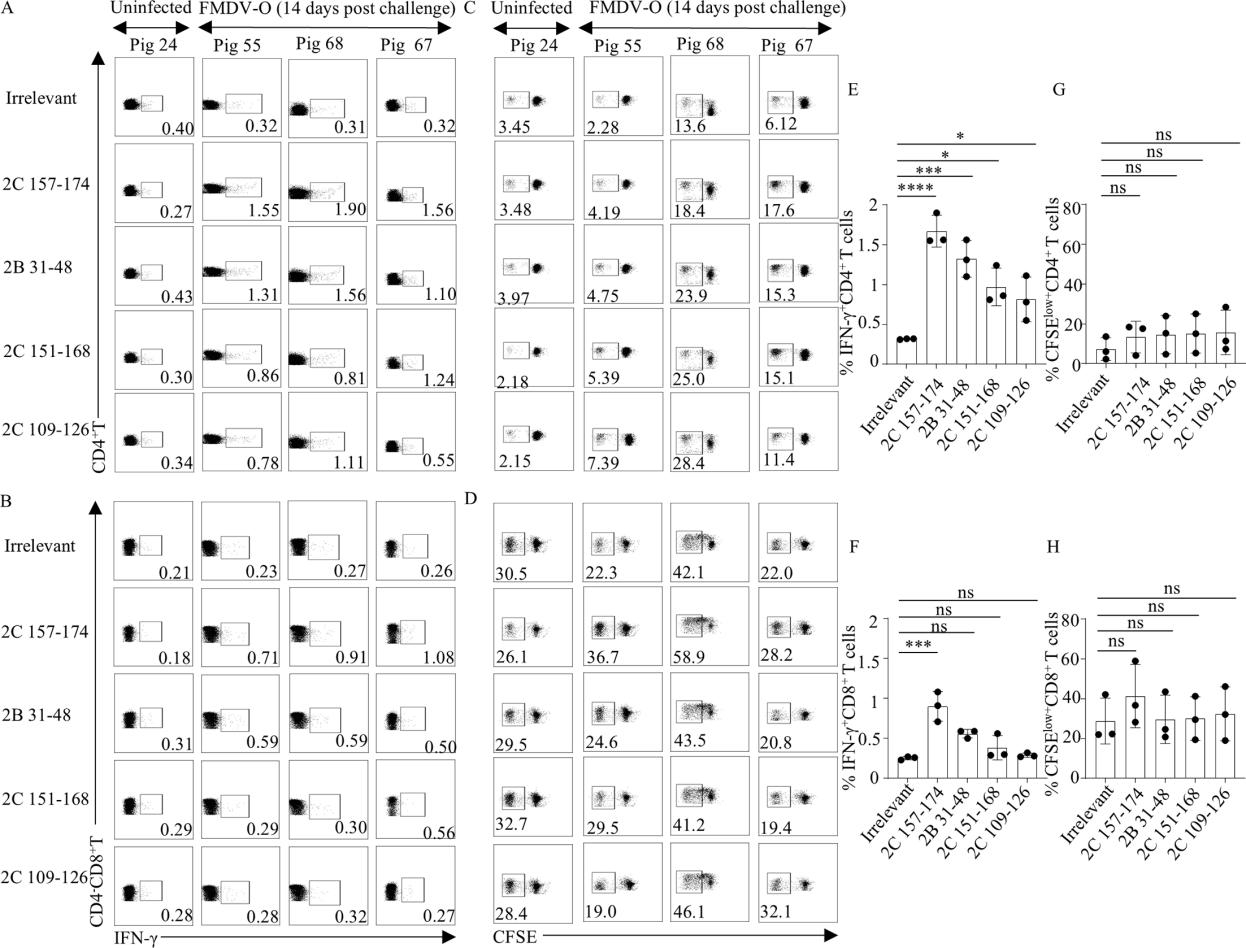
Detection of peptide-induced T-cell phenotypes by ICS and CFSE staining. PBMCs were isolated from FMDV-infected or uninfected pigs at 14 days post-infection. PBMCs were stimulated with individual peptides that showed IFN-γ reactivity as identified by ELISpot, followed by ICS and CFSE staining. (**A and B**) Representative dot plots of peptide-induced IFN-γ secretion in CD4^+^ and CD8^+^ T cells. (**C and D**) Representative dot plots of peptide-induced proliferation of CD4^+^ and CD8^+^ T cells. (**E and F**) Percentages of IFN-γ⁺ CD4⁺ and CD8⁺ T cells following *in vitro* peptide stimulation. (**G and H**) Percentages of proliferating CD4⁺ and CD8⁺ T cells in response to peptide stimulation, determined by CFSE. ICS and CFSE results from the FMDV O challenge group are presented as the mean ± SD (*n* = 3). Statistical significance was assessed by one-way ANOVA, ns: not significant, *P < 0.05, **P < 0.01, ***P < 0.001, ****P < 0.0001, significantly different from the Irrelevant control.

To further characterize the phenotypes and functions of peptide-specific T cells, ICS was performed (Fig. S1; Fig. 3A and B). Considering CD8α is expressed not only on classical cytotoxic T lymphocytes (CTLs) but also on other immune cells, including γδ T cells, NK cells, and certain CD4^+^CD8α^+^ T cells. We excluded TCR γδ^+^T cells, CD3^-^CD4^-^CD8α^+^NK cells, and CD3^+^CD4^+^CD8α^+^T cells through flow cytometry gating strategies. The results showed that all four peptides specifically activated CD4^+^ T cells to secrete IFN-γ. Notably, 2C 157–174 also activated CD8^+^ T cells, suggesting potential presence of CD4^+^ and CD8^+^ T-cell epitopes within this sequence (Fig. 3B, E and F). Similarly, CFSE-based proliferation assay revealed that peptides 2B 31–48, 2C 109–126, 2C 151–168, and 2C 157–174 more stimulated CD4^+^ or CD8 T-cell proliferation in PBMCs from FMDV-infected pigs compared to the control group (Fig. 3C, D, G and H), although the difference between the groups was not statistically significant (*P* > 0.05). But we observed a potential tendency to promote a proliferation in cell numbers, which is consistent with the results in ICS.

### SLA restriction and conservation analysis of identified T-cell epitopes

**Fig 4:**
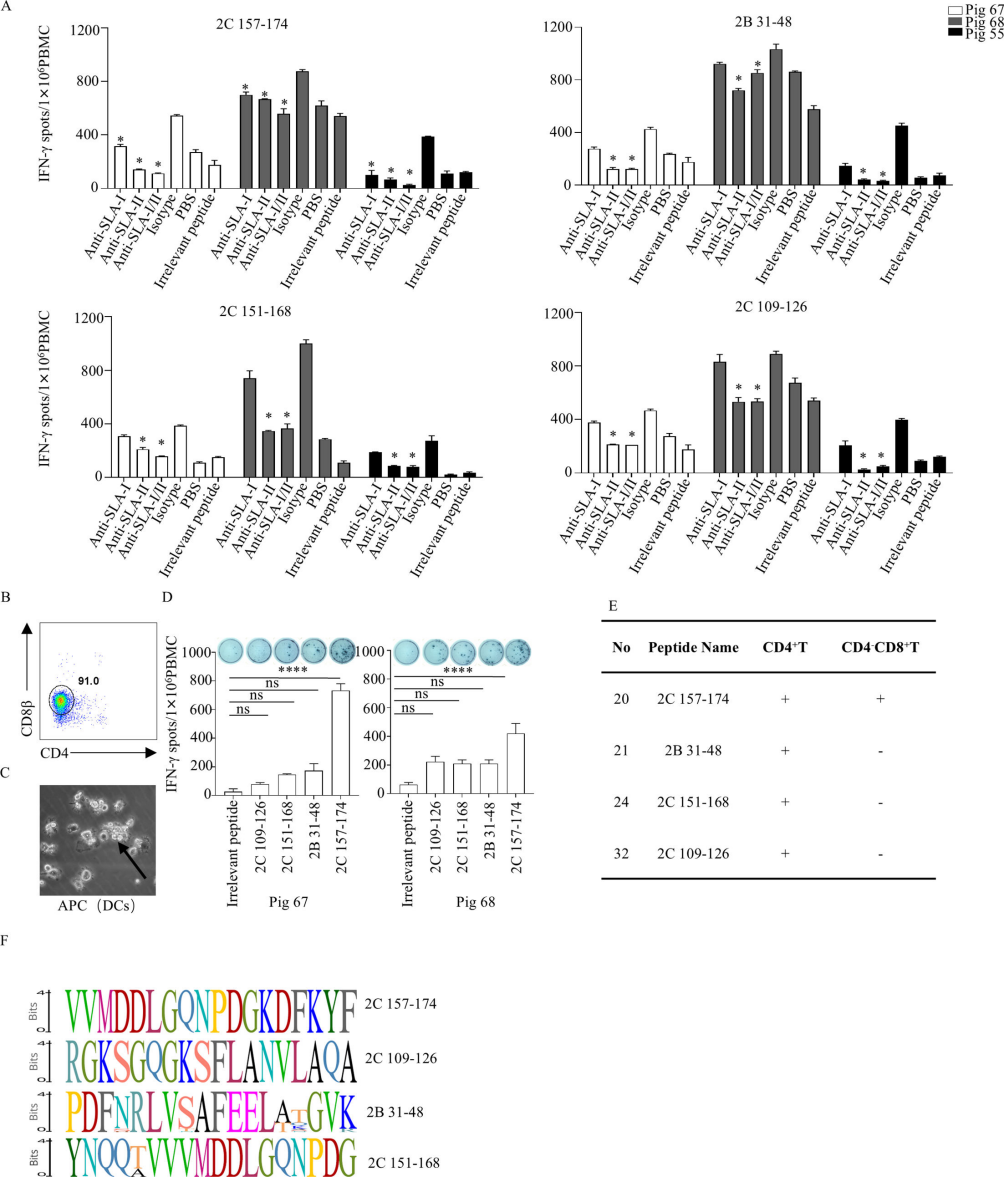
Analysis of SLA restriction and conservation of T-cell epitopes. (**A**) Inhibitory effect of porcine SLA I/II Abs on the proliferative response to peptides 2B and 2C. PBMCs were isolated from FMDV-infected pigs at 14 days post-infection, treated with SLA I/II Abs (15 μg/well), and the responses were quantified by IFN-γ ELISpot. An irrelevant peptide was used to evaluate the specificity of peptide-induced responses. An isotype control Ab was used to examine the specificity of restriction by anti-SLA Abs, compared to the isotype Ab in the presence of anti-SLA Abs. (**B**) PBMCs from FMDV-infected pigs #67 and #68 were magnetically sorted with anti-CD8β monoclonal Ab, and flow cytometry was used to determine the purity of CD8β. (**C**) Monocyte-derived dendritic cell culture; black arrows indicate cells with dendrites. (**D**) CD8β^+^ T cells and antigen-presenting cells were added to IFN-γ ELISpot plates at a ratio of 10:1, and T-cell epitopes of 2C and 2B were added for IFN-γ detection. (**E**) Reactivity of different peptides in inducing CD4⁺ T cells and CD4⁻CD8⁺ T cells. (**F**) Conservation analysis of 2B and 2C peptides; the logo plot was generated by aligning sequences of 2B and 2C from seven FMDV serotypes. Letters represent amino acid codes, and heights are proportional to frequencies. Repeat three times for each sample (*n* = 3), and the data are presented as the mean ± standard deviation. The data were assessed by one-way ANOVA, ns: not significant. *P < 0.05, **P < 0.01, ***P < 0.001, ****P < 0.0001, significantly different from the isotype or irrelevant peptide control.

To determine SLA restriction of the identified epitopes, antigen presentation was blocked *in vitro* using specific antibodies against SLA class I or class II molecules (21). IFN-γ ELISpot results indicated that blocking with anti-SLA-II antibodies caused a greater reduction of peptide-specific IFN-γ-secreting cells (IFN-γ SCs) than with anti-SLA-I antibodies (Fig. 4), suggesting that the peptide recognition by T cells is primarily restricted by SLA-II. Notably, peptide 2C 157–174-specific I IFN-γ SCs were also significantly reduced by anti-SLA-I antibodies (Fig. 4A), confirming the presence of the CD8^+^ T-cell epitope (Fig. 3).

To confirm whether peptide 2C 157–174 specifically activated classical CD8αβ T cells in pigs, CD8β^+^ T cells were isolated from the PBMCs of FMDV-infected pigs and co-cultured with Mo-DCs in the presence of the peptide. The results showed that peptides 2C 157–174 induced CD8β^+^ T cells to secrete IFN-γ (Fig. 4B through D), confirming their capacity to activate classical CD8^+^ T cells rather than CD4^+^CD8α^+^ memory/effector T cells or CD8α^+^ NK cells.

Sequence alignments of the peptides across seven FMDV serotypes revealed high conservation in the four peptides (Fig. 4E; Table S5). Among them, peptides 2C 157–174 and 2C 109–126 showed 100% identity across all serotypes. Peptide 2B 31–48 exhibited 72.2% identity, with variations mainly in serotype O (three variations) and SAT 1-3 (five variations), while peptide 2C 109–126 had 94.4% identity, varying predominantly in SAT 1-3 (Fig. 4E). All four peptides were highly conserved in currently prevalent serotypes A and O.

Given the prevalence of serotypes A and O FMDV in cattle and pigs in China (32), cross-species T-cell reactivities of the four-epitope identified in pigs were evaluated in cattle. The results showed that only peptide 2C 109–126 and 2C 157–174 elicited IFN-γ responses of bovine T cells (Fig. S3).

### Design and characterization of the Trimer-CD40L-TB vaccine

**Fig 5:**
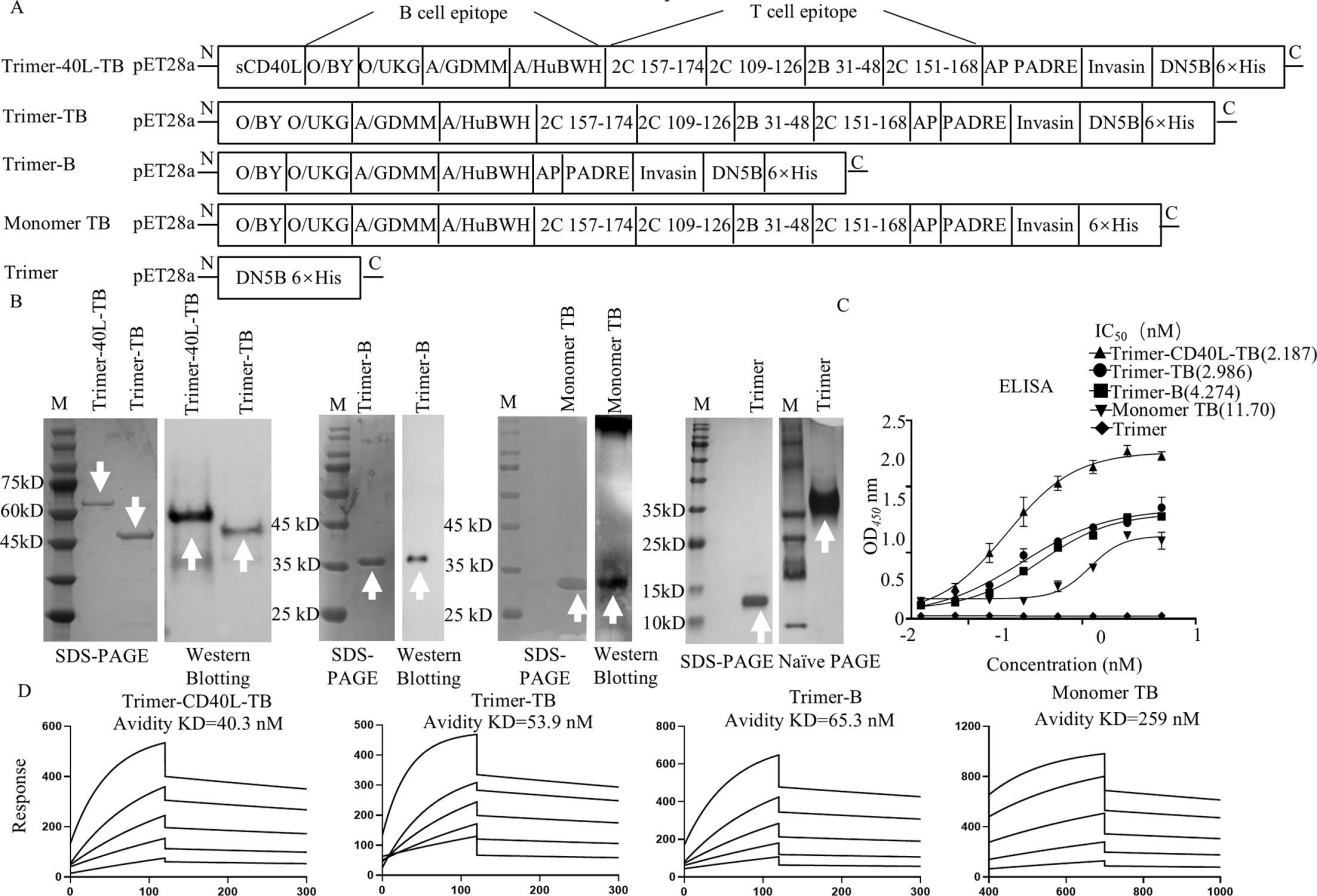
Construction and characterization of CD40L-fused multi-epitope trimeric proteins. (**A**) Trimer-40L-TB expresses a trimeric fusion protein containing the extracellular domain of CD40L and TB epitopes. 5B-TB encodes a fusion protein lacking CD40L. 5B-B encodes a fusion protein lacking CD40L and T epitopes. Monomer TB encodes a monomeric vaccine, and 5B serves as a trimerization motif without antigenic epitopes. Numbers indicate the expected molecular weights of proteins in each construct. (**B**) SDS-PAGE and western blotting analysis of protein expression, identified using FMDV-positive serum. (**C**) ELISA detection of the reactivity of five proteins. Equal concentrations of the five proteins were coated, serially diluted, and detected using biotinylated O8 single-domain Ab. Repeat three times for each sample (*n* = 3), and data are presented as the mean ± standard deviation. (**D**) SPR analysis of the binding affinity of trimeric and monomeric proteins. O8 single-domain Ab was immobilized on an M5 sensor chip, and four proteins were applied as analytes. Note: B-cell epitopes: VP1: 137–156 (O/BY; O/Panasia; A/GDMM; A/HW); sCD40L: residues 113–261; T-cell epitopes: 2C [157–174], 2C [151–168], 2C [109–126], and 2B [31–48]; AP: RRRWCKPPP; 5B: artificially designed protein self-assembling trimerization motif.

Previous studies have shown that targeting DCs for antigen delivery can elicit potent T-cell responses (14), and CD40L served as an effective DC-targeting and activating molecule (33). Therefore, we designed a novel fused protein vaccine, Trimer-CD40L-TB (Fig. 5A), integrating the extracellular domain of CD40L, four identified T-cell epitopes (one SLA I-restricted CD8^+^ T-cell epitope and three SLA II-restricted CD4^+^ T-cell epitopes), four B-cell epitopes (derived from the GH loops of two serotype O and two serotype A strains), a cell-penetrating peptide (to facilitate intracellular delivery), and a self-assembling trimerization domain (to promote multimerization) (Fig. S4). Genes encoding Trimer-CD40L-TB, Trimer-TB (lacking CD40L), Trimer-B (B cell epitopes only), Monomer TB (monomeric form), and Trimer (empty trimer control) were cloned into pET-28a, expressed in *E. coli* BL21(DE3), and purified by nickel-affinity chromatography. SDS-PAGE and western blot analysis confirmed the sizes of each recombinant protein (~65, ~48, ~35, and ~18 kDa, respectively) and specific reactivity with FMDV-positive serum (Fig. 5B). As no specific antibody was available for artificial 5B protein (B-cell epitope module), its trimeric (~45 kDa) and monomeric (~15 kDa) forms were verified under native and denatured conditions, respectively (Fig. 5B).

To assess whether the trimeric proteins retained native conformational epitopes, we performed ELISA and SPR using the single-domain antibody O8 (34), which recognizes a conformational GH loop. Monomer TB served as a control. ELISA results showed that stronger O8 binding by the trimeric proteins (Trimer-CD40L-TB IC_50_= 2.187, Trimer-TB IC_50_= 2.986, Trimer-B IC_50_= 4.274) compared with Monomer TB (IC_50_= 11.70) (Fig. 5C). SPR further confirmed that higher binding affinity of Trimer-CD40L-TB (KD = 40.3 nM), Trimer-TB (KD = 53.9 nM), and Trimer-B (KD = 65.3 nM) relative to Monomer TB (KD = 259 nM) (Fig. 5D), demonstrating that the trimers presented B-cell epitopes in their native conformations.

**Fig 6:**
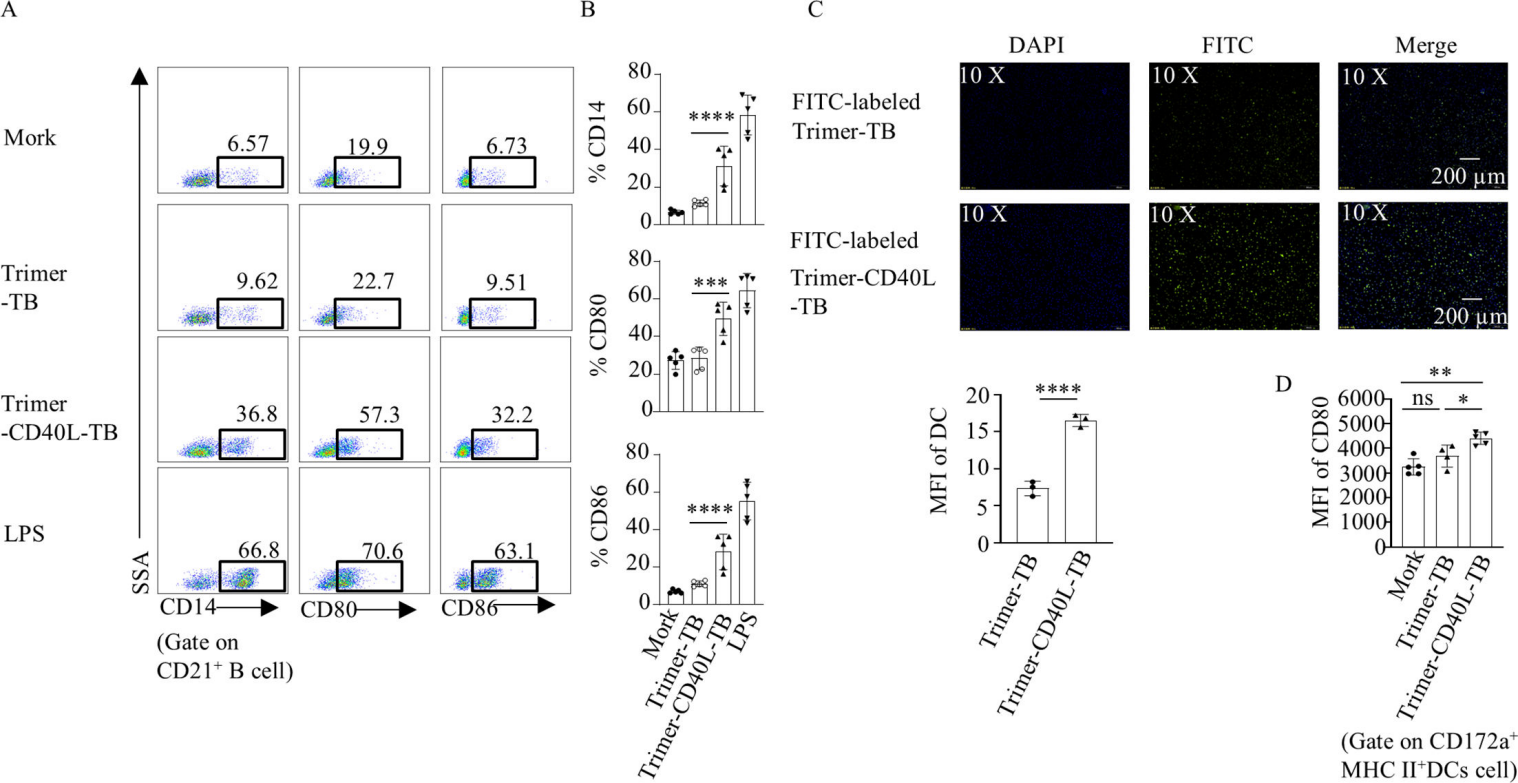
*In vitro* bioactivity detection of Trimer-CD40L-TB. Equal amounts of Trimer-CD40L-TB, Trimer-TB, and DMEM were incubated with porcine PBMCs, and the activation of B cells and monocyte-derived dendritic cells (Mo-DCs) was detected by flow cytometry. (**A**) Representative dot plots of CD21^+^ B-cell activation. (**B**) Differences in the expression levels of CD80, CD86, and CD14 in CD21^+^ B cells (*n* = 5). (**C**) Differential analysis of Mo-DCs phagocytosing FITC-labeled Trimer-CD40L-TB and Trimer-TB proteins (*n* = 3). (**D**) Differences in mean fluorescence intensity of CD80 in Mo-DCs (*n* = 5). Data are presented as mean ± standard deviation. ns: not significant. *P < 0.05, **P < 0.01, ***P < 0.001, ****P < 0.0001.

Given that CD40-CD40L interaction enhances cytokine secretion, co-stimulatory molecule expression, and antigen cross-presentation (35), we evaluated the activation capacity of Trimer-CD40L-TB on CD40^+^ cells. Flow cytometry revealed that Trimer-CD40L-TB significantly upregulated CD80, CD86, and CD14 on CD21^+^ B cells compared to the blank control and Trimer-TB groups (Fig. 6A and B), confirming its ability to bind and activate CD40^+^ cells. When incubated with porcine Mo-DCs, FITC-labeled Trimer-CD40L-TB was taken up more efficiently than Trimer-TB by the Mo-DCs, suggesting that the fusion of CD40L promoted antigen uptake by DCs (Fig. 6C). Moreover, when gating on CD172a^+^ MHC II^hi^ Mo-DCs (excluding CD163^+^, CD21^+^, and CD14^+^ cells; Fig. S2B), Trimer-CD40L-TB treatment significantly upregulated CD80 expression compared to Trimer-TB (Fig. 6D), enhancing DC maturation.

### Trimer-CD40L-TB vaccine elicits robust cellular immunity and promotes memory T-cell development

**Fig 7:**
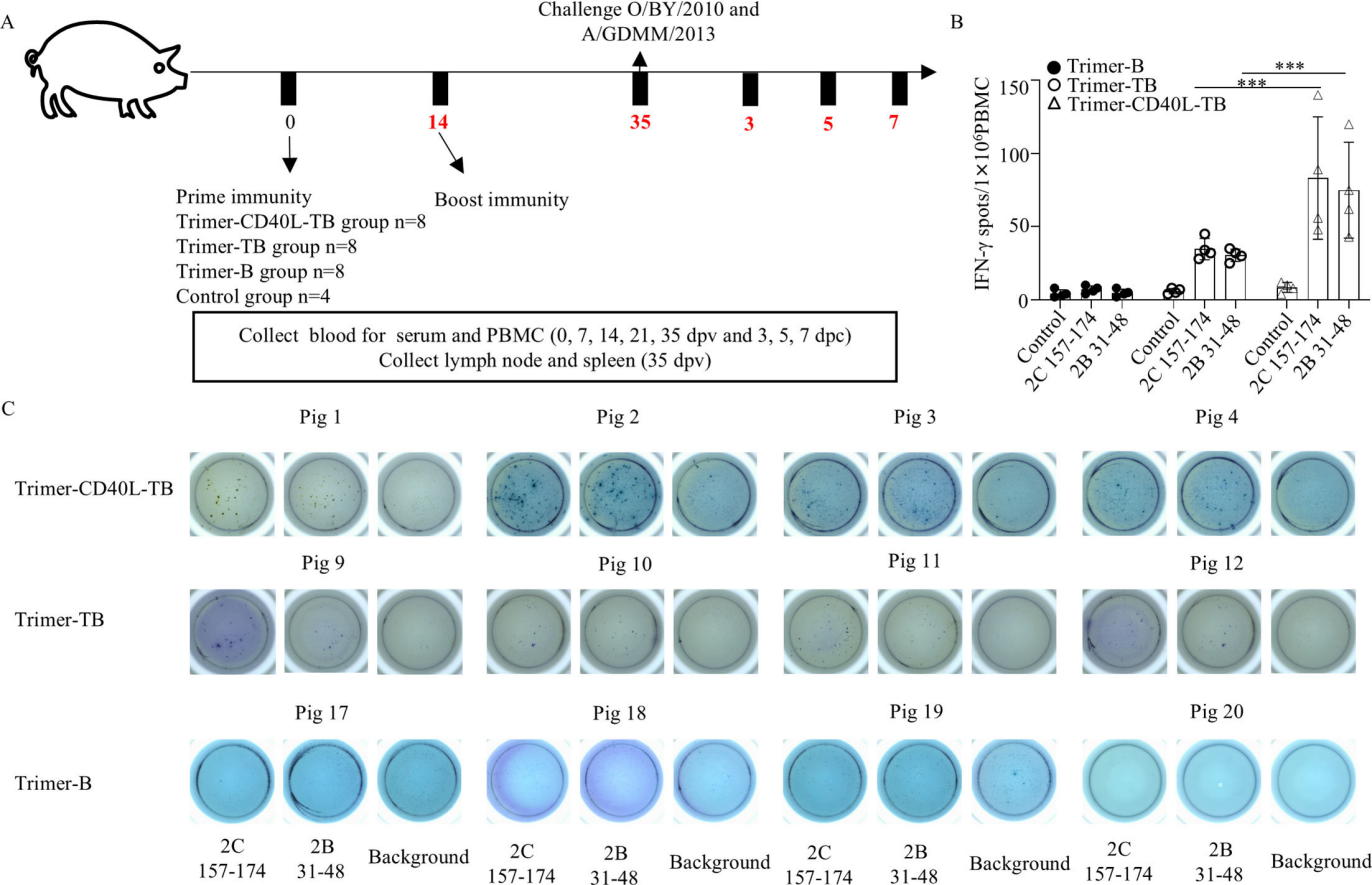
Animal experiment design and activation analysis of T-cell epitopes. Four groups of pigs were immunized with Trimer-CD40L-TB (*n* = 8), Trimer-TB (*n* = 8), Trimer-B (*n* = 8), and Trimer (*n* = 4), followed by a booster immunization on 14 dpv and a challenge protection experiment on 35 dpv. PBMCs were isolated from each group at 35 dpv and stimulated with or without 2B and 2C peptides for 36 h, and IFN-γ-producing T cells were identified with the ELISpot assay. (**A**) Schematic diagram of the animal experiment design and sample collection. (**B**) Comparison of the total number of IFN-γ-secreting cells per million PBMCs in each group. (**C**) Representative IFN-γ spots in each group. Data are presented as the mean ± standard deviation (*n* = 4). The data were assessed by two-way ANOVA, ns: not significant, *P < 0.05, **P < 0.01, ***P < 0.001, ****P < 0.0001, significantly different from the Trimer-TB group.

**Fig 8:**
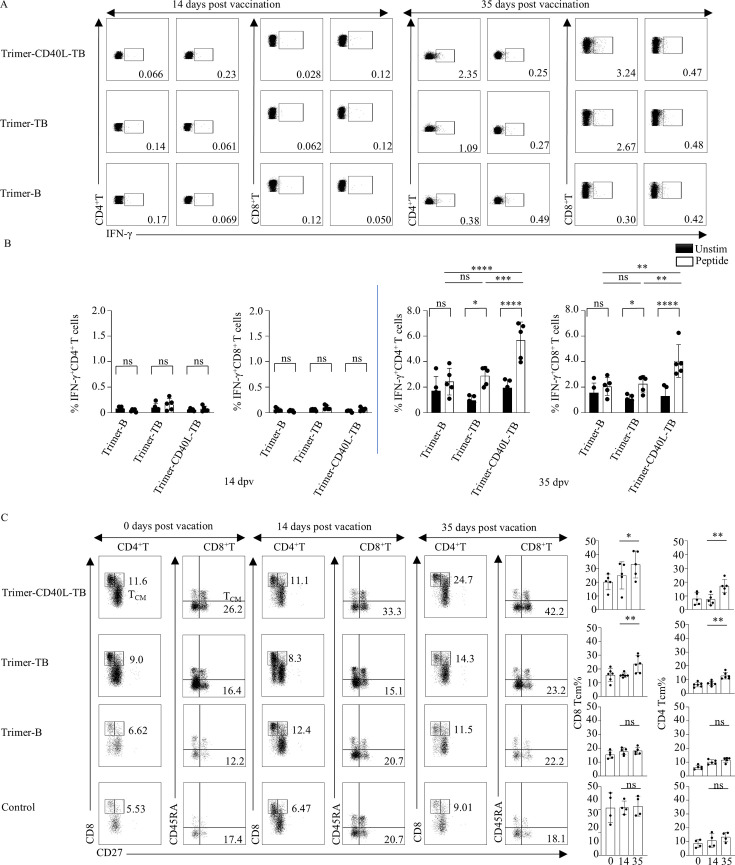
FMDV-specific IFN-γ-producing T cells produced by vaccinated pigs and the development of memory T cells. PBMCs were isolated from each group at 14 and 35 dpv, restimulated with or without 2B and 2C peptides for 10 h, and IFN-γ-producing T cells were detected by intracellular cytokine staining. (**A**) Representative dot plots of IFN-γ-producing CD4^+^ and CD8^+^ T cells in each group after restimulation with or without peptide pool (2C 157–174, 2B 31–48, 2C 109–126, and 2C 151–168). (**B**) Comparison of the percentages of FMDV-specific IFN-γ-producing CD4^+^ and CD8^+^ T cells in each group (*n* = 5). ICS assay is presented as the mean ± standard deviation (*n* = 5). (**C**) Effector and memory CD4^+^ and CD8^+^ T cells in PBMCs of each group were identified by multicolor flow cytometry, and the percent changes among 0, 14, and 35 dpv were compared. The left panel shows representative dot plots of CD4^+^ T_CM_ (CD4^+^CD27^+^CD8α^+^) and CD8^+^ T_CM_ (CD45RA⁻CD27^+^) detected in each group. The right panel shows bar graphs depicting the percentage differences between CD4^+^ T_CM_ and CD8^+^ T_CM_. T_CM_ assay is presented as the mean ± standard deviation (*n* = 4 or 5). All data were assessed by two-way ANOVA, ns: not significant, *P < 0.05, **P < 0.01, ***P < 0.001, ****P < 0.0001.

Pigs were immunized intramuscularly twice at 2-week intervals with Trimer-CD40L-TB, Trimer-TB, Trimer-B, or Trimer emulsified in adjuvant 201 (1:1) (Fig. 7A). To evaluate whether each T-cell epitope in the fusion proteins was presented, peptide-specific IFN-γ SCs in the PBMCs collected 35 dpv were detected by IFN-γ ELISpot upon re-stimulation with single peptides. Out of the four epitopes, 2C 157–174- and 2B 31–48-specific IFN-γ SCs were detected (Fig. 7B and C). The frequency of IFN-γ SCs in the Trimer-CD40L-TB group was significantly higher than that in the Trimer-TB group, consistent with the trend of IFN-γ-producing T cells between the two groups detected by ICS (Fig. 8A and B), indicating that the fused CD40L enhanced the immunogenicity of specific T-cell epitopes. However, the other two peptides 2C 151–168 and 2C 109–126 induced IFN-γ production only in pigs 6, 13, and 14 (Fig. S5). SLA-II genotyping revealed that pigs 6, 13, and 14 had SLA II alleles different from those of other immunized pigs but similar to pigs 67, 68, and 55 used for initial screening.

After boost immunization, FMDV-specific T-cell responses were evaluated at 35 dpv (Fig. 8A). Both the Trimer-CD40L-TB and Trimer-TB groups showed significant antigen-specific IFN-γ^+^CD4^+^ and IFN-γ^+^CD8^+^ T-cell response, with higher magnitude in the former group (CD4^+^: 2.8% vs 1.45% on average; CD8^+^: 4.2% vs 2.67% on average) (Fig. 8A and B), further demonstrating the effect of CD40L on enhanced T cell responses.

To further assess the breadth of peptide-specific T cells, PBMCs isolated from each group at 35 dpv were cultured and restimulated *in vitro* with or without T-cell peptides. The levels of IFN-γ, IL-2, TNF-α, and IL-4 in the supernatant were measured by ELISA (Fig. S6). The results indicated that the levels of IL-2, IFN-γ, and TNF-α secreted by peptide-specific T cells were highest in the Trimer-CD40L-TB group, then lower in the Trimer-TB group, and least in the Trimer-B group. However, no IL-4 was detected among all the groups at this time point (Fig. S6).

Considering the importance of central memory T cells (T_CM_) in resisting viral infections (36) and the fact that targeting antigen to DCs promotes T_CM_ differentiation (14). T_CM_ subsets in each group were analyzed and compared at 35 dpv using porcine-specific markers (CD4^+^ T_CM_: CD27^+^CD8α^+^; CD8^+^ T_CM_: CD27^+^CD45RA^+^, gating strategy in Fig. S1). The results showed that only the Trimer-CD40L-TB and Trimer-TB groups had significantly higher percentages of CD4^+^ (number mean 17.22 vs 12.95) and CD8^+^ T_CM_ (number mean 32.82 vs 23.62) (Fig. 8C), indicating that the fusion of T-cell epitopes and CD40L in the vaccines can enhance T_CM_ differentiation.

The CD40L-CD40 signal promotes Th1 immune response (25, 37). To verify this, T-bet (a Th1/CTL marker) and Ki67 (proliferation marker) were co-stained in T cells (gating strategy in Fig. S1). The Trimer-CD40L-TB group showed significant increases in CD4^+^T-bet^+^Ki67^+^ (Th1) and CD8^+^T-bet^+^Ki67^+^ (CTL) cells from 14 dpv to 35 dpv, whereas no change was observed in the other three groups (Fig. S7A and B), highlighting the role of CD40L in promoting Th1/CTL expansion.

### Trimer-CD40L-TB vaccine induces potent humoral immunity and confers serotypes A and O FMDV protection in pigs

The CD40L-CD40 signaling pathway is known to promote B-cell proliferation (25, 37); therefore, we examined proliferative B cells in each group. The proportion of CD79α^+^Ki67^+^ B cells significantly increased from 14 dpv to 35 dpv in both the Trimer-CD40L-TB and Trimer-TB groups but not in the Trimer-B group (Fig. S7B). These results illustrated that the integration of CD40L and T-cell epitopes into a vaccine promoted B-cell proliferation and may have a synergistic effect when both are incorporated (like in Trimer-CD40L-TB).

**Fig 9:**
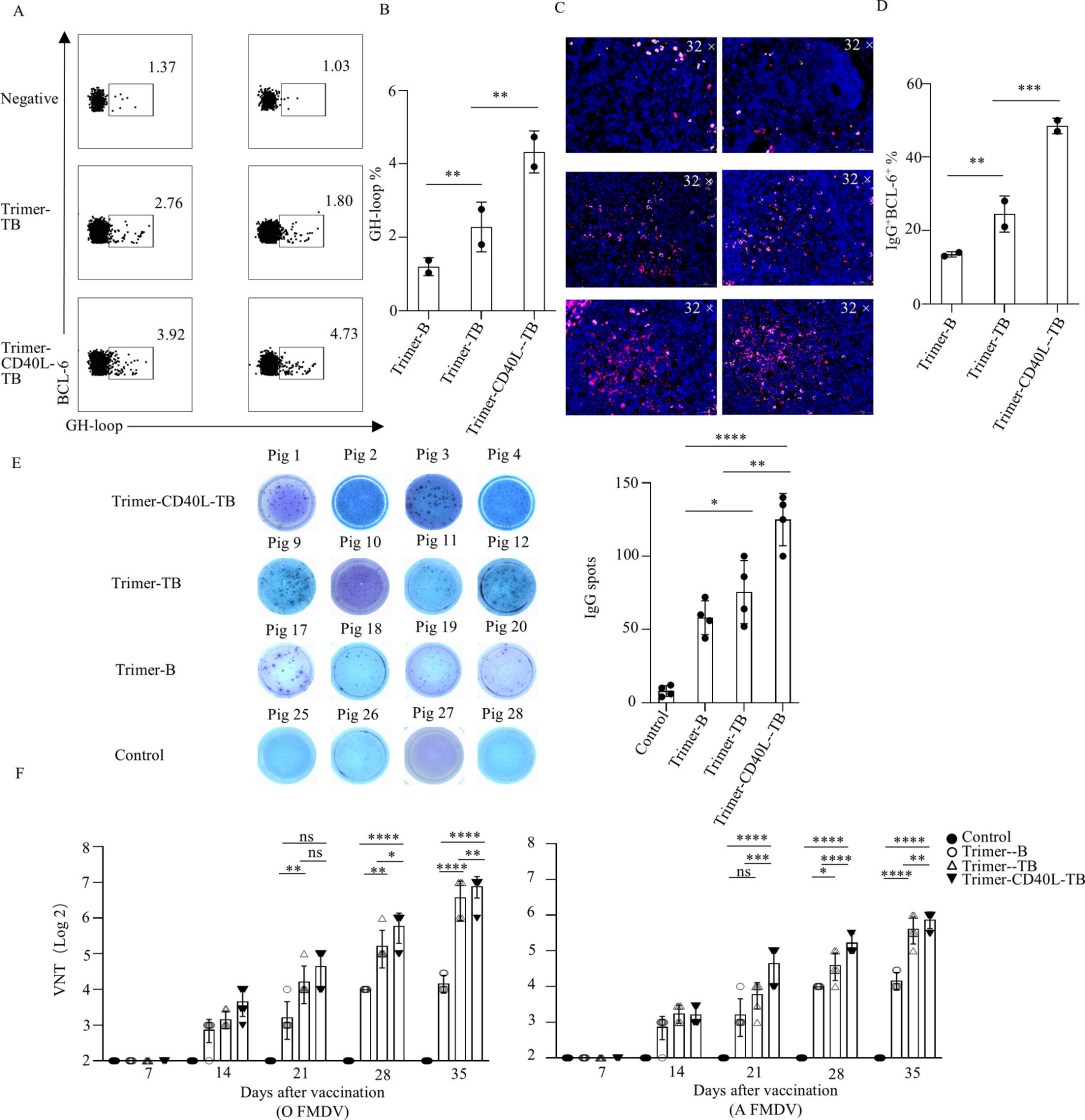
Analysis of Trimer-CD40L-TB vaccine-induced FMDV GH-Loop-specific GC B-cell counts, IgG Abs, neutralizing Abs, and accompanying broad-spectrum protection against FMDV serotypes O and A. Two pigs from each of the Trimer-CD40L-TB, Trimer-TB, and control groups were euthanized at 35 dpv, and lymphoid tissues were collected. (**A**) Isolating single cells was assessed by flow cytometry. Representative dot plots of GH-Loop-specific GC B cells in the Trimer-CD40L-TB, Trimer-TB, and control groups (*n* = 2). (**B**) Bar graph analyzing the differences in the number of GH-Loop-specific GC B cells among the three groups (*n* = 2/group). (**C**) Fluorescence staining analysis of GC B cells in tissue sections, with BCL-6 (red) and IgG (purple) used to localize B cells in GC. (**D**) Analysis of differences in the number of IgG^+^BCL-6^+^ fluorescent cells among the three groups (*n* = 2/group). (**E**) PBMCs were isolated from pigs at 35 dpv. After coating with anti-porcine IgG Ab, PBMCs were added and incubated for 24 h, then biotinylated Trimer-B protein was used to detect the number of IgG antibodies secreted by B cells specific to B epitopes (*n* = 4/group). (**F**) Serum samples were collected from each group of pigs, and FMDV NAb titers were detected by a neutralization test. FMDV NAb titers among the four groups were compared at 7, 14, 21, 28, and 35 dpv (*n* = 4/group). All data assay is presented as the mean ± standard deviation (*n* = 2 or 4). All data were assessed by one or two-way ANOVA, ns: not significant, *P < 0.05, **P < 0.01, ***P < 0.001, ****P < 0.0001.

Germinal centers (GCs) are essential for the generation of high-affinity antibodies and memory B cells, with CD40L playing a central role in GC formation (38). To further evaluate whether T-cell epitopes and CD40L could enhance humoral response induced by B-cell epitopes, we examined GH loop-specific GC B cells in each group by flow cytometry. The results showed that the Trimer-CD40L-TB group had a higher proportion of GH loop-specific GC B cells than the Trimer-TB group at 35 dpv, though the latter also induced more GC B cells than did the Trimer-B group (Fig. 9A and B). Immunofluorescence staining of IgG/BCL-6 in lymph nodes further confirmed that CD40L and T-cell epitopes acted synergistically to promote GC formation in the Trimer-CD40L-TB group (Fig. 9C and D).

Further detection of FMDV-specific antibody-secreting cells (ASCs) by IgG ELISpot assay showed that the Trimer-CD40L-TB group had the highest number of FMDV-specific ASCs, followed by Trimer-TB and Trimer-B group (Fig. 9E). Similarly, the Trimer-CD40L-TB group also had the highest NAb titers against serotypes O and A (mean = 22–32) at 21 dpv, followed by the Trimer-TB (mean = 13.8–18.6) and the Trimer-B group (mean = ~9.3) (Fig. 9F). By 35 dpv, the fastest rise and the highest NAbs titers were observed in the Trimer-CD40L-TB group compared to the other groups (Fig. 9F). These findings demonstrated that the fusion of CD40L and T-cell epitopes can synergistically enhance Trimer-CD40L-TB-induced humoral immunity.

**Fig 10:**
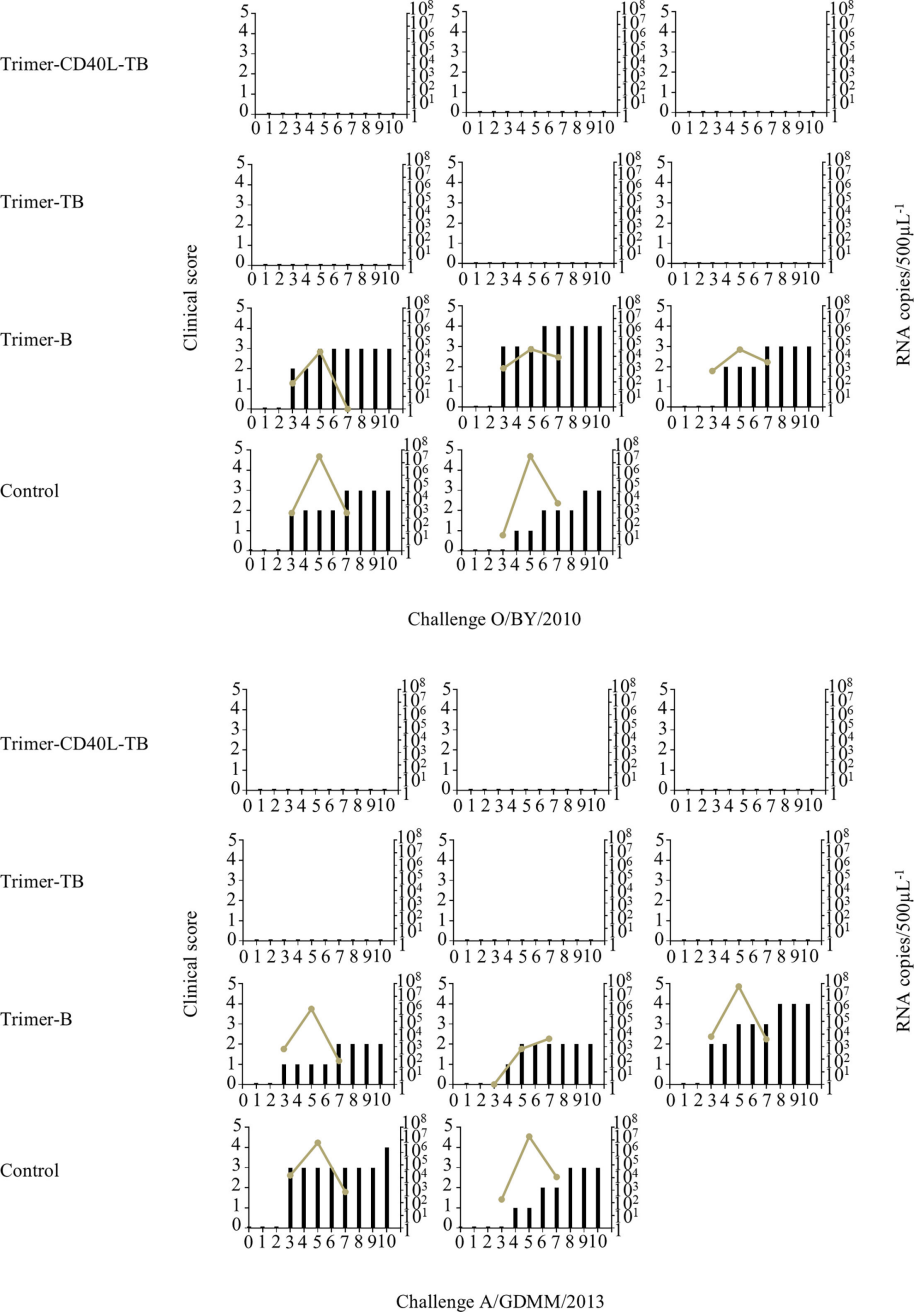
The comparison of clinical scores and viremia in each group of pigs from day 0 to day 10 after the challenge with FMDV O/BY/2010 and A/GDMM/2013. The left *Y*-axis indicates the clinical index. The right *Y*-axis indicates viral loads in sera (dashed line).

Based on the potent memory T-cell responses and high NAb titers induced by the Trimer-CD40L-TB at 35 dpv, a challenge experiment was performed using FMDV strains O/BY and A/GD. Both the Trimer-CD40L-TB and Trimer-TB groups provided 100% protection, with immunized pigs showing no clinical symptoms such as viremia or foot-and-mouth lesions (Fig. 10). No protection was observed in the groups lacking T-cell epitopes, underscoring the essential role of the identified T-cell epitopes in the induction of high-level NAb responses and complete cross-protection against prevalent serotype O and A FMDV strains.

## DISCUSSION

Cellular immunity is crucial for enhancing the efficacy of FMD vaccines. However, the limited identification of porcine Th cell and CTL epitopes has hindered the incorporation of T-cell targeted strategies into FMD vaccine design (20, 39), thus impeding a deeper understanding of their immune mechanisms. In this study, we systematically characterized the kinetics of T-cell responses to NSPs 2B and 2C in FMDV-infected pigs and identified immunodominant CD4^+^/CD8^+^ T-cell epitopes. By integrating these epitopes into a DC-targeted multi-epitope delivery platform, we developed a broad-spectrum trimeric vaccine, Trimer-CD40L-TB. Through a vaccination-challenge experiment in pigs, we demonstrated that the combined action of T-cell epitopes and CD40L synergistically enhances memory T-cell differentiation, GC formation, and the production of NAbs. These findings provide a substantive experimental foundation for developing novel, highly effective, and broad-spectrum FMD vaccines.

To minimize the potential influence of porcine SLA polymorphism on epitope presentation, pigs from multiple litters were included in the experimental design to ensure representation of diverse genetic backgrounds. Although FMDV NSPs may contribute to host immune suppression during viral replication (40), this study observed that the IFN-γ^+^ T-cell response to the 2B/2C peptide pool peaked at 14 dpc (Fig. 1B and C), coinciding with the viral clearance phase. This temporal association suggests that these T-cell responses may participate in establishing effective antiviral immunity. Although the response weakened by 28 dpc, it remained significantly above baseline levels, consistent with the transition of effector T cells into memory pools (41).

Based on the observed response kinetics, where 14 dpc represented the effector phase and 28 dpc the memory phase, this study employed an orthogonal peptide pool screening strategy combined with IFN-γ ELISpot to identify four core epitopes (peptide 2B 31–48, 2C 109–126, 2C 151–168, and 2C 157–174) within the 2B/2C protein. Notably, multiple reactive epitopes clustered in adjacent regions of the 2C protein, such as 109–126 and 151–168/157–174, suggest the presence of an “epitope cluster” (42). This spatial arrangement may improve antigen presentation efficiency and facilitate cross-recognition by T-cell receptors (43), thereby amplifying the overall immune responses.

Further analyses using flow cytometry, including ICS and CFSE assays (Fig. 3), revealed that peptide 2C 157–174 activated both CD4^+^ and CD8^+^ T cells, which indicates that it may be a bispecific epitope. This property is of significant antiviral relevance since CD4^+^ T cells assist in B-cell antibody production and macrophage activation (44), whereas CD8^+^ T cells mediate direct killing of infected cells (45). Such dual functionality may stem from the presence of binding motifs for both MHC class I and class II molecules within the peptide sequence. Antibody blocking experiments (Fig. 4A) confirmed that peptide 2B 31–48, 2C 109–126, and 2C 151–168 are SLA-II restricted and mainly activate CD4^+^ T cells (46), while 2C 157–174 is dually restricted by SLA-I/II, consistent with the profile of a bispecific epitope. Although the ICS gating strategy has excluded most non-CTL cell populations, there are still a small number of CD8αα^+^T cells present (47). Porcine CD4^-^CD8^+^αβ T cells are also referred to as cytolytic T cells (CTL), for which CD8β serves as a specific marker. To this end, we performed CD8β^+^ T-cell sorting followed by DC co-culture. The results (Fig. 4B through D) verified that peptide 2C 157–174 specifically activates these classical CD8^+^ T cells, thereby ruling out contributions from non-classical CD8αα^+^-expressing subsets.

Sequence alignment across multiple FMDV serotypes showed that peptide 2C 109–126 and 2C 157–174 are 100% conserved among the seven major serotypes, identifying them as ideal candidates for broad-spectrum vaccine design. Furthermore, 2B 31–48 and 2C 151–168 are highly conserved in the currently prevalent serotypes (A/O), supporting their practical applicability. Cross-species immunogenicity evaluation further demonstrated that all four peptides induce IFN-γ responses in pigs infected with either serotypes A or O, while only peptides 2C 109–126 and 2C 157–174 induced detectable responses in cattle (Fig. S3A through D). This divergence is likely attributable to differences in MHC presentation between species—SLA versus bovine leukocyte antigen (BoLA) (48). The findings suggest that the epitopes identified in this study may have limited applicability in cattle, underscoring the need to identify bovine-specific T-cell epitopes in future studies.

Based on the dominant T-cell epitopes identified in this study, we designed a DC-targeting trimeric vaccine, Trimer-CD40L-TB (Fig. 5A), which contains two key innovative features. First, immunogenicity was enhanced through trimer-driven structural stabilization. Inspired by the self-assembling trimeric scaffold DN5B, which has been successfully employed in vaccine designs for Lassa virus GP1/GP2 (49) and RSV-F protein (50), we applied this strategy for the first time in the context of FMDV vaccines. Non-denaturing electrophoresis confirmed that DN5B facilitates stable trimer formation (Fig. 5B). Moreover, evaluation using an antibody specific for a conformational epitope of FMDV VP1 GH loop revealed that trimerization increased antigen-binding affinity by twofold to threefold (Fig. 5C and 6D). This structural enhancement enhances the efficiency of vaccine interaction with DCs and B cells, providing a solid foundation for potent immune activation. Second, the vaccine leverages CD40L-mediated targeting to synergistically activate APCs. The CD40-CD40L signaling pathway not only promotes APC maturation but also enhances antigen cross-presentation, thereby facilitating CD8^+^ T-cell activation (51). In our construct, CD40L was incorporated at the N-terminus of the antigen. This design offers significant biological advantages: the N-terminal CD40L domain actively engages CD40 on the surface of DCs/B cells, while the C-terminal trimer domain presents the T/B tandem epitopes in a stabilized conformation. This configuration significantly enhances DC phagocytosis and upregulates the expression of co-stimulatory molecules (CD80/CD86) (Fig. 6).

Notably, while the Trimer-CD40L-TB vaccine successfully induced IFN-γ^+^ T-cell responses targeting the peptides 2C 157–174 and 2B 31–48 (Fig. 7B), the responses to peptides 2C 151–168 and 2C 109–126 were relatively limited. One possible reason is that the two epitopes (2C 152–168; 2C 110–128) fused to Trimer-CD40L-TB were not generated correctly during antigen processing in the proteasome. This often happens and is a challenge faced by multi-epitope-based vaccines based on previous reports (8, 52). Another reason T cells mainly recognize and bind MHC class I or II molecules through their T-cell receptor (TCR), we speculate that the above differences may be attributed to the different MHC-binding affinities between epitope sequences or the limited diversity of MHC between pigs, which restricts the correct presentation of T-cell epitopes (21, 53, 54). As shown in Fig. S5 and Table S6, we observed that variations in SLA II haplotypes led to differential recognition of the same epitope across different pigs. This aligns with reports on PRRSV nanoparticle vaccines and HIV vaccines, in which immunodominance hierarchies are shaped by host genetic background (53, 54). As for MHC-binding affinity, it has been shown that MHC binding affinity of an epitope, along with TCR recognition, affects its immunogenicity (55, 56). While direct measurement of MHC-binding affinity would provide valuable insights in human and mouse studies, such analysis faces substantial challenges in porcine immunology due to a lack of detailed SLA haplotype and recombinant SLA proteins. Given the current lack of high-throughput peptide-binding affinity validation techniques for porcine SLA alleles, this study was unable to directly determine the binding affinity between candidate epitopes and specific SLA molecules. Therefore, the underlying molecular mechanisms underlying the observed differences in MHC-related immune responses, such as precise MHC restriction, are still speculative. Together, these findings highlight the importance of incorporating detailed SLA haplotyping data into future epitope optimization strategies.

The function of Th1 CD4^+^ T-cell subsets plays a pivotal regulatory role in FMDV protective immunity (57). T-bet, a nuclear transcription factor specifically expressed in Th1 cells, mediates antiviral immune responses via the secretion of cytokines such as IFN-γ, IL-2, and TNF-α (58). Previous studies have indicated that incorporating T-cell epitopes or adjuvants that enhance cellular immune responses can improve the capacity of FMD vaccines to elicit early and sustained immunity—a phenomenon potentially mediated by T-cell-derived cytokines, including IFN-γ, IL-2, and TNF-α (10, 11, 59, 60). In our study, the epitopes 2C 157–174 and 2B 31–48 were found to induce the production of IFN-γ, IL-2, and TNF-α in immunized pigs (Fig. S6). These results not only corroborate the contribution of T-cell responses to improved vaccine efficacy but also highlight the highly efficient DC-targeting strategy mediated by CD40L.

Beyond confirming the role of T-cell epitopes and CD40L-targeting strategy in improving T-cell responses, this study provides mechanistic insight into how T cells support B-cell immunity. Vaccines containing T-cell epitopes and CD40L significantly enhanced the proliferation of Ki67^+^ B cells, increased germinal center (GC) B-cell frequency, elevated antigen-specific IgG secretion (Fig. S7; Fig. 9), and boosted serum NAb levels (Fig. 9E). These effects can be attributed to a coordinated mechanism: CD40L binding to CD40 on dendritic cells (DCs) upregulates costimulatory molecules CD80/CD86 (Fig. 6A and B) and enhances antigen phagocytosis (Fig. 6C), thereby facilitating antigen presentation to T cells. Finally, it should be noted that all immune response was evaluated only at 35 dpv. Although the observed T_CM_ differentiation and increased germinal center (GC) B-cell frequency suggest potential for long-term immunity, the durability of protection remains to be validated in follow-up studies involving immunological evaluation and challenges at extended timepoints (e.g., 6 or 12 months) to assess real-world applicability.

The genetic and antigenic diversity of diverse FMDV serotypes leads to the lack of cross-protection, which is a major obstacle to develop broad-spectrum vaccines. In this study, the Trimer-CD40L-TB vaccine, which incorporates the bifunctional epitope 2C 157–174 along with the highly conserved epitope 2B 31–48, conferred complete protection in pigs against challenge with both serotype A (GDMM/2013 strain, epidemic strains in China) and serotype O (BY/2010 strain, epidemic strains in China) (Fig. 10). In contrast, the Trimer-B vaccine, which contains only B-cell epitopes, did not confer protection. It should be clarified, however, that while *in vitro* peptide screening data suggest reactivity in cattle, *in vivo* protective efficacy remains to be verified, particularly given known MHC disparities (SLA versus BoLA) between species. Furthermore, as the challenge experiments utilized one representative strain per serotype, generalizability to other topotypes or lineages requires further evaluation. Future studies should include a wider panel of challenge strains to more comprehensively assess the vaccine’s breadth. Nonetheless, these findings underscore the value of integrating CD40L and well-selected T-cell epitopes, particularly bifunctional epitopes such as 2C 157–174, and offer a promising strategy for achieving cross-serotype protection against FMDV.

In conclusion, this study developed a novel vaccine, Trimer-CD40L-TB, by identifying immunodominant CD4^+^/CD8^+^ T-cell epitopes from FMDV NSPs 2B/2C, and incorporating several key elements: a DN5B scaffold, CD40L extracellular domain, and a multi-epitope integration. This design significantly enhances antigen stability, promotes DC activation, and strengthens T-cell responses. Although the presentation efficiency varied among individual epitopes, the core vaccine concept offers an innovative strategy for developing FMDV vaccines with broad-spectrum coverage and sustained protective immunity. In addition, cross-species protection remains unproven, and future studies are needed to validate *in vivo* efficacy in cattle, considering MHC differences between species. Therefore, future studies should focus on clarifying the mechanisms of epitope processing, validating the function of memory T cells, and improving cross-species adaptability to facilitate the clinical translation of this vaccine platform.

## MATERIALS AND METHODS

### Cells and viruses

BHK-21 cells were cultured in Dulbecco’s modified Eagle’s medium (DMEM; Sigma-Aldrich Corporation, St. Louis, MO, USA) supplemented with 10% fetal bovine serum (Gibco, Invitrogen Corporation, Carlsbad, CA, USA), 3 mM glutamine, penicillin (100 IU/mL), and streptomycin (50 µg/mL) (Gibco, USA). Foot-and-mouth disease virus (FMDV) strains O/BY/2010 and A/GDMM/2013 were stored by the World Organization for Animal Health/National Foot and Mouth Disease Reference Laboratory (Lanzhou, China).

### The overlapping peptides library of NSPs 2B and 2C protein

In this study, 75 peptides of 18 amino acids (Table S1) in length were prepared, covering FMDV NSPs 2B and 2C. All peptide sequences were designed based on the O/BY/2010 strain (GenBank Accession No.: JN998085) and synthesized by GenScript Biotech Corporation (Nanjing, China) with a purity of ≥85%. As shown in Table S2, the 75 peptides were grouped into 19 peptide pools using an orthogonal design for screening T-cell epitopes. Individual peptides were dissolved at a concentration of 1 mg/mL, while peptide pools or mixtures were prepared at 100 µg/mL. The peptide solutions were stored at ˗−80°C.

### Animal challenge experiments for screening T-cell epitopes

FMDV-seronegative pigs and cattle, as determined by liquid-phase blocking ELISA, were selected for challenge experiments conducted in the Animal Biosafety Level 3 Laboratory at the Lanzhou Veterinary Research Institute.

For the initial screening of T-cell epitopes, seven 3-month-old pigs were allocated into two groups: five were intramuscularly inoculated with 10⁵ ID₅₀ of FMDV O/BY/2010, with the remaining two receiving PBS as controls.

To assess the breadth of T-cell epitopes across viruses and species, the animals were divided into the following groups. Fifteen 3-month-old pigs were allocated such that five were intramuscularly inoculated with 10⁵ ID₅₀ of FMDV O/BY/2010, five were inoculated with 10⁴ ID₅₀ of FMDV A/GDMM/2013, and five were administered PBS as controls. Similarly, eight 3-month-old cattle were divided into three groups: three were inoculated via the tongue with 10³ ID₅₀ of FMDV A/GDMM/2013, three were inoculated with FMDV O/BY/2010, and two received PBS as controls.

All animals were monitored daily for clinical signs for 10 days post-challenge (dpc). PBMCs were isolated from blood samples collected at 7, 14, and 28 dpc using species-specific PBMC isolation kits (Solarbio, China).

### Swine IFN-γ ELISpot assay

The porcine IFN-γ ELISpot assay was performed according to the kit instructions and previous reports (61). Briefly, nitrocellulose plates (Millipore, USA) were coated overnight at 5 µg/mL with an anti-porcine IFN-γ monoclonal antibody (mAb; pIFNγ-I, Mabtech, Sweden) in PBS. Then, 2 × 10^5^ PBMCs were seeded per well and stimulated with peptides at a final concentration of 5 µg/mL for 36 h at 37°C. After washing with PBST (PBS containing 0.05% Tween 20), plates were incubated for 2 h at room temperature with biotinylated anti-porcine IFN-γ mAb (P2C11, Mabtech, Sweden) at 0.5 µg/mL. Following five washes with PBS, HRP-conjugated streptavidin was added and incubated for 1 h. After another five washes, spots were developed using TMB substrate for 10–30 min. The reaction was stopped by rinsing with deionized water, and the plates were air-dried before spot enumeration using an AID ELISpot reader (AID, Germany). Results were standardized as IFN-γ-secreting cells per million cells.

To assess the role of major histocompatibility complex (MHC) in peptide-induced IFN-γ secretion, blocking experiments were performed using anti-porcine SLA class I (mAb 74-11-10, IgG2b) and class II (mAb MSA-3, IgG2a) antibodies, which were previously shown to antagonize SLA recognition (21, 62). PBMCs were pre-incubated with each mAb (150 μg/well) in anti-IFN-γ-coated ELISpot plates for 12 h at 37°C. Peptides were then added at a final concentration of 10 μg/mL, and cells were cultured for an additional 36 h. Subsequent washing and detection steps were carried out as described above.

To further evaluate the contribution of CD8β^+^ T cells, PBMCs from FMDV-infected pigs (#67 and #68) were magnetically sorted using porcine CD8β-FITC antibody (Thermo Fisher Scientific, USA) at a 1:50 dilution, with sorting purity confirmed by flow cytometry. Monocyte-derived dendritic cells (Mo-DCs) were generated as previously described (63, 64). Briefly, isolated PBMCs were plated for 3 h, after which non-adherent cells were removed. Adherent monocytes were cultured for 7 days in complete RPMI-1640 medium supplemented with 30 ng/mL GM-CSF and 10 ng/mL IL-4 (Abcam, UK), with a medium replaced at half volume on days 3, 5, and 7. On day 7, Mo-DCs were harvested and counted. These antigen-presenting cells (APCs) were then co-cultured with sorted CD8β^+^ T cells at a 1:10 ratio (APC: T cell) in IFN-γ ELISpot plates in the presence of 2B or 2C T cell epitopes. IFN-γ-secreting T cells were detected as outlined above.

### CFSE staining

PBMCs were isolated as previously described and labeled with carboxyfluorescein succinimidyl ester (Horizon CFSE; BD Biosciences, USA) to track proliferation. Briefly, a 10 mM CFSE stock solution was prepared in Dulbecco’s phosphate-buffered saline (DPBS). After washing with DPBS to remove serum components, cells were resuspended at 1–3 × 10⁷ cells/mL and stained with CFSE for 10 min in a 37°C water bath. The reaction was quenched by three washes with DPBS. Labeled cells were then rested for 12 h in a 37°C, 5% CO₂ incubator and subsequently stimulated with peptides and porcine IL-2 (Abcam) for 72 h. Finally, cells were harvested, stained for surface markers CD3, CD4, and CD8, fixed in 0.5% paraformaldehyde, and analyzed by flow cytometry to assess T-cell proliferation based on CFSE.

### Intracellular cytokine staining

For ICS, 2 × 10⁶ cells per well were plated in 96-well round-bottom plates and stimulated with or without T-cell epitope peptides (5 μg/mL) for 24 h, with brefeldin A added for the final 6 h. Subsequently, 1 × 10⁶ cells were transferred to 96-well V-bottom plates, stained with fixable viability dye eFluor 780 (FVD-780) (Thermo Fisher Scientific) according to the manufacturer’s instructions, washed twice, and surface markers were stained on ice (Table S3). After fixation and permeabilization using a commercial kit (BD Biosciences), cells were incubated with APC-conjugated anti-porcine IFN-γ antibody (clone P2G10) at room temperature for 30 min. Stained cells were washed, resuspended in PBS, and analyzed by flow cytometry.

### Conservative analysis

Full-length amino acid sequences of FMDV 2B and 2C proteins from swine and cattle isolates were retrieved from the NCBI database (Table S5). Multiple sequence alignment was performed using Geneious Prime software (version 2022.0.2). Sequence conservation within the identified epitope regions was subsequently visualized as logo plots using the same software.

### SLA II genes analysis

The total cellular DNA from PBMCs was extracted using MiniBEST Genomic DNA Extraction Kit (Takara, Japan) according to the manufacturer’s instructions. The extracted total DNAs were stored at −80°C for later use. The full SLA-II DRA, DRB, DQA, and DQB gene coding sequences were amplified directly from the total DNAs extracted from PBMCs using the primeSTAR Max DNA polymerase (Takara, Japan) according to the manufacturer’s instructions. PCR primers and amplification reactions set published previously (65, 66).

### Preparation of recombinant proteins

The DC-targeted immune-enhancing protein Trimer-CD40L-TB was designed to provide broad-spectrum protection against FMDV serotypes O and A (Fig. 1). The recombinant protein included the extracellular domain of porcine CD40L (Uniprot: Q95MQ5, aa 113-261) (27, 67), T-cell epitopes from the 2B (aa 31–48) and 2C (aa 109–126, 151–168, and 157–174) proteins, B-cell epitopes derived from FMDV strains O/BY/2010 (JQ973889), O/UKG/2001 (AJ539141), A/GDMM/2013 (KF450794), and A/HuBWH/2009 (JF792355), the DN5B trimerization motif (68), the delivery molecule AP, the immune-enhancing peptide PADRE, and the universal T-cell epitope Invasin (11). These elements were connected by a “GGSGG” linker (Fig. 5A; Fig. S4). The codon-optimized gene was synthesized and cloned into the pET-28a vector by GenScript Biotech (China).

For expression, the recombinant plasmid was transformed into *E. coli* BL21 (DE3) cells (TransGen Biotech, China). A single colony was used to inoculate a large-scale culture, which was induced with 1 mM IPTG at an OD_600_ of 0.8 and further cultured at 16°C for 16 h. After induction, cells were harvested by centrifugation and lysed by sonication. SDS-PAGE analysis of the lysate confirmed that the target proteins (Trimer-CD40L-TB, Trimer-TB, Monomer TB, Trimer-B, and Trimer) were predominantly expressed as inclusion bodies. The inclusion bodies were isolated, washed with TE and urea-containing buffers, and solubilized in Buffer A (8 M urea, 2.5 mM DTT). Target proteins were purified by nickel-affinity chromatography using imidazole elution, dialyzed against glycine buffer (pH 7.4), quantified, and stored at –70°C.

### Functional characterization of recombinant proteins

The immunogenicity of the purified proteins was evaluated by Western blot. Proteins were electrophoresed on 12% SDS-PAGE, transferred onto nitrocellulose membranes, and primed with serum from FMDV-recovered pigs (diluted 1:5,000), followed by incubation with an HRP-conjugated anti-pig secondary antibody. After washing, bands were visualized using an ECL kit (Sigma-Aldrich, USA) according to the manufacturer’s instructions.

To assess whether the recombinant proteins Trimer-CD40L-TB, Trimer-TB, and Trimer-B present the B-cell epitopes in their native conformation, surface plasmon resonance (SPR) was performed using an O8 nanobody (specifically targets the conformational GH loop of FMDV) immobilized on a CM5 chip (GE Healthcare, Chicago, IL, USA), with the recombinant proteins as analytes. In brief, the serially diluted Trimer-CD40L-TB, Trimer-TB, Trimer-B, and Monomer TB concentrations (ranging from 2.5 to 50 nM, prepared by twofold serial dilution in running buffer) flowed over the NAb (O8)-immobilized CM5 sensor chip surface using a multi-cycle kinetics approach. All sensor grams shown are reference-subtracted and solvent-corrected. Binding kinetics were analyzed by fitting the data to a 1:1 binding model using the Biacore T200 evaluation software. In addition, to avoid mass-transport limitations, the mass transfer coefficient ($t_{c}$) must exceed the reaction rate constant (k⋅a) by a factor of 100 or more.

Similarly, an indirect ELISA was conducted using the O8 nanobody as the primary detection reagent: recombinant proteins (Trimer B 1 µg/mL, 10 nM; Trimer-TB 1.5 µg/mL, 10 nM; Trimer-CD40L-TB 1.95 µg/mL, 10 nM) were coated onto plates overnight at 4°C. The plates were incubated with 50 μL of a twofold serial dilution of the biotinylated nanobodies (starting concentration: 0.8 μg/mL, 50 nM) at 37°C for 1 h. Subsequently, HRP-conjugated streptavidin (Sigma-Aldrich Corporation) was added for 1 h at 37°C. Then, the plates were stained with 3,3′,5,5′-tetramethylbenzidine (TMB). The absorbance at 450 nm (OD_450_) was measured, and the half-maximal inhibitory concentration (IC_50_) was calculated using GraphPad Prism.

To examine CD40L-mediated immune activation, porcine PBMCs were cultured for 16 hours with Trimer-CD40L-TB (10 µg/mL), Trimer-TB (10 µg/mL), LPS, or PBS. Cells were subsequently stained with fluorescently labeled antibodies directed against DC markers (CD172a, CD163, CD80, SLA-II, CD14) and B-cell markers (CD21, CD80, CD86, and CD14; detailed in Table S3), and analyzed by flow cytometry to determine activation status.

For cellular uptake assays, Mo-DCs (1 × 10^6^) were cultured in six-well plates and treated with 10 μM FITC-labeled Trimer-CD40L-TB for 6 h. After washing with PBS, cells were fixed with 4% paraformaldehyde, permeabilized with 0.1% Triton X-100, and stained with 4′,6-diamidino-2-phenylindole (DAPI). Fluorescence images were acquired using a fluorescence microscope. By Image J, soft processing image was to obtain the average fluorescence intensity.

### Vaccination and FMDV challenge in pigs

Twenty-eight 8- to 9-week-old pigs from an FMDV-free farm were randomly divided into four groups: Trimer-CD40L-TB (*n* = 8, numbered Pigs 1–8), Trimer-TB (*n* = 8, numbered Pigs 9–16), Trimer-B (*n* = 8, numbered Pigs 17–24), and Trimer (*n* = 4, numbered Pigs 25–28). Each recombinant protein (1 mg/mL) was emulsified with Montanide ISA-201 adjuvant (Seppic, France) at a 1:1 ratio using an IKTA emulsifier to form a water-in-oil vaccine. All pigs received a primary intramuscular immunization with 3 mL (1 mg) of the respective vaccine, followed by a booster with the same dose on day 14.

At 35 days post-vaccination (dpv), two pigs from each of the Trimer-CD40L-TB, Trimer-TB, and Trimer-B groups were euthanized for lymph node collection to analyze GC B cells. The remaining animals were challenged at 35 dpv: one group was inoculated with 3 mL of 1,000 ID_50_ FMDV strain O/BY/2010 (O/mya 98), and the other with 3 mL of 100 ID_50_ strain A/GDMM/CHA/2013. Clinical signs were recorded daily from 1 to 10 dpc, and the final protection rate was calculated.

For the viral load assay, viral RNAs were extracted from the sera samples using Trizol reagent (Takara) according to the instructions; the cDNA was synthesized using PrimeScript RT Master Mix. Using a probe based on the FMDV 3D gene sequence published by OIE (14). The CFX96 Touch Real-Time PCR Detection System (Bio-Rad, Hercules, CA, USA) was used for virus quantification.

### Immunophenotype analysis

Surface marker staining was conducted to characterize porcine T-cell subsets and memory phenotypes, as previously described (14, 69). Briefly, 2 × 10^6^ PBMCs were stained on ice for 30 min with 50 μL of a cocktail of fluorochrome-labeled antibodies against porcine surface markers, including CD3, CD4, CD8α, γδT, CD45RA, CCR7, and CD27. Detailed information on all mAbs used is provided in Table S3.

### Intranuclear transcription factor staining

Intranuclear staining for Ki-67, T-bet, CD79α, and BCL-6 was performed as previously described (14). Following surface marker staining, cells were fixed and permeabilized using the eBioscience Foxp3/Transcription Factor Staining Buffer Set (Invitrogen, USA). They were then incubated with antibodies specific for Ki-67, T-bet, CD79α, or BCL-6. After washing, cells were resuspended in PBS and analyzed by flow cytometry. Details of mAbs are summarized in Table S3.

### Cytokine testing

Cells were suspended in complete RPMI 1640 medium at a density of 2 × 10^6^ cells/mL and stimulated with or without peptide pool (2C 157–174, 2B 31–48, 2C 109–126, and 2C 151–168) (15 μg/mL) in round- bottomed 96- well plates at 300 μL/well, 37°C, and 5% CO_2_ for 24 h. Cytokines in the supernatants were detected using porcine IFN-γ, TNF-α, IL-2, and IL-4 ELISA Kit (Absin, China), according to the manufacturer’s instructions.

### Tissue immunofluorescence assay

Lymph node samples were fixed in formalin, embedded in paraffin, and sectioned at 3-μm thickness. Sections were dewaxed in xylene and rehydrated through a graded ethanol series. For immunofluorescence co-staining of IgG and BCL-6, slides were first incubated overnight at 4°C with purified goat anti-pig IgG (1:200, clone AAI40, Bio-Rad), followed by donkey anti-goat IgG HRP (1:200, clone A-21124, Sigma-Aldrich) for 45 min at room temperature and then TYR-690 for 30 min. Subsequently, slides were incubated overnight at 4°C with purified mouse anti-Bcl-6 (1:100, clone K112-91, BD Biosciences), followed by goat anti-mouse IgG HRP (1:200, Thermo Fisher Scientific) for 45 min and TYR-620 dye for 30 min. Nuclei were counterstained with DAPI. Slides were scanned using a digital slide scanner (P250 FLASH, Jinan Dandery Electronics, China) and analyzed with a data image analysis system (Halo 101-WL-HALO-1, Indica Labs, China).

### Swine IgG ELISpot

To quantify vaccine-specific antibody-secreting cells (ASCs), PVDF ELISpot plates (Millipore) were activated with 25% ethanol, washed thoroughly with distilled water, and coated overnight with FMDV B epitope (20 μg/mL) at 100 μL/well. After blocking with RPMI-1640 containing 10% porcine serum, porcine PBMCs were added at 3 × 10^5^ cells/well in triplicate and cultured for 16 h at 37°C in 5% CO_2_. Plates were then washed, incubated with biotinylated anti-pig IgG (5 µg/mL, Mabtech) for 2 h at room temperature, followed by streptavidin-HRP for 1 h. Spots were developed using TMB substrate (Mabtech), and the reaction was stopped by rinsing with deionized water. After air-drying, spots were counted using an AID ELISpot reader (AID, Germany). Results were standardized as IgG-secreting cells per million cells.

### Micro-neutralization assay

Virus-neutralizing antibody (NAb) titers against FMDV were determined by micro-neutralization assays as previously described (34). Briefly, serum samples were heat-inactivated at 56°C and subjected to twofold serial dilutions starting from 1:4. Each dilution was mixed with 100 TCID_50_ of FMDV strain O/BY/2010 or A/GDMM/2013 and incubated at 37°C for 1 h. The serum–virus mixture was then transferred to monolayers of BHK-21 cells in 96-well plates. After adsorption at 37°C for 1 h, the medium was replaced with DMEM containing 2% FBS. Cells were cultured for 72 h, and cytopathic effects (CPE) were recorded. The NAb titers of each sample were calculated using the Karber method.

### Statistical analysis

All statistical analysis was performed using GraphPad Prism (version 9.5.0, USA). For comparisons among multiple groups, one-way or two-way ANOVA was applied, followed by Bonferroni’s post-test. Results were considered statistically significant at *P < 0.05, **P < 0.01, ***P < 0.001, ****P < 0.0001.

## SUPPLEMENTAL MATERIAL

Supplemental material mbio.03586-25-s0001.docx Fig. S1-S7; Tables S1-S6.

ASM does not own the copyrights to Supplemental Material that may be linked to, or accessed through, an article. The authors have granted ASM a non-exclusive, world-wide license to publish the Supplemental Material files. Please contact the corresponding author directly for reuse.
