# Supplementary material for: T cell responses to nonstructural proteins promote cross-serotype immunity to foot-and-mouth disease virus
Source: mBio. 2026 Apr 6;17(5):e03586-25. doi: 10.1128/mbio.03586-25 (PMC13170233; doi:10.1128/mbio.03586-25)
Supplement: Supplemental material — Fig. S1-S7; Tables S1-S6. [file mbio.03586-25-s0001.docx]

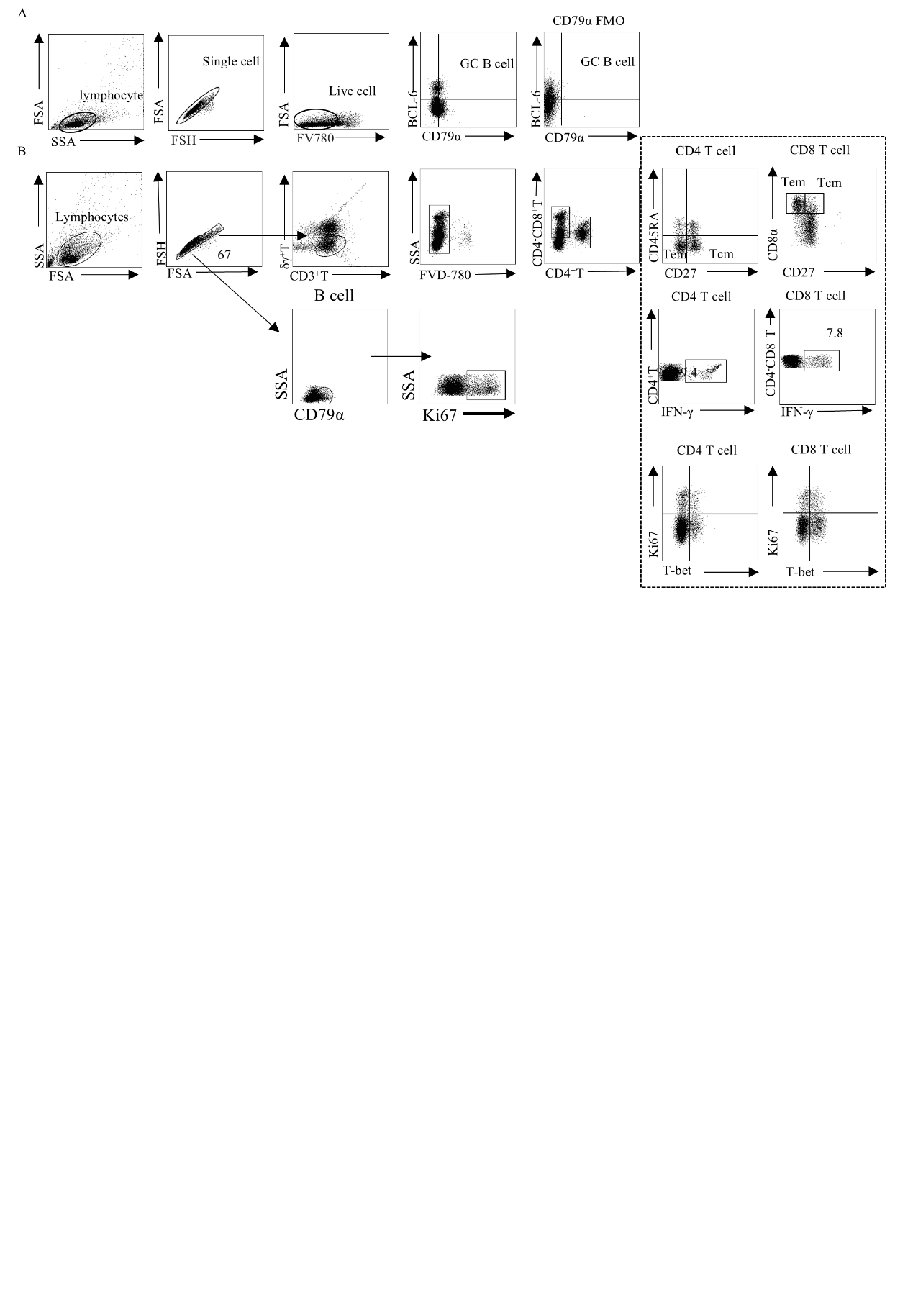


Supplementary **Fig. 1** Flow cytometry analysis of gating strategies for CD4^+^, CD8^+^T and CD79α^+^Bcells. (A) Gating strategy for distinct GC B cell. GC B cell were identified as CD79α^+^BCL6^+^ and CD79α^+^ fluorescence minus one control (FMO) (B) Gating strategy for distinct T cell subsets. Lymphocytes were gated according to FSC-A/SSC-A properties; doublets and dead cells were excluded (Fixable Viability Dye eFluor™ 780). γδ T cells were identified on lymphocytes and excluded. CD4^+^ and CD8^+^ T cells were identified as TCRγδ^-^CD3^+^CD4^+^ and TCRγδ^-^CD3^+^CD4^-^CD8α^+^, respectively. B cell were identified as CD79α^+^ and representative dot-plots of Ki67-expressing on CD79α^+^ B cell. Furthermore, CD4 T_CM_ (CD4^+^CD27^+^CD8α^+^), T_EM_ (CD4^+^CD27^-^CD8α^+^) and CD8 T_CM_ (CD45RA^-^CD27^+^), T_EM_ (CD45RA^-^CD27^-^) were identified on CD4^+^ and CD8^+^ T cell. For IFN-γ, representative dot-plots of IFN-γ-expressing CD4^+^ T and CD8α^+^ T upon mitogen stimulation. Down figure Representative dot-plots of ki67-positive T-bet^+^CD4^+^ T and CD8α^+^ T cell.


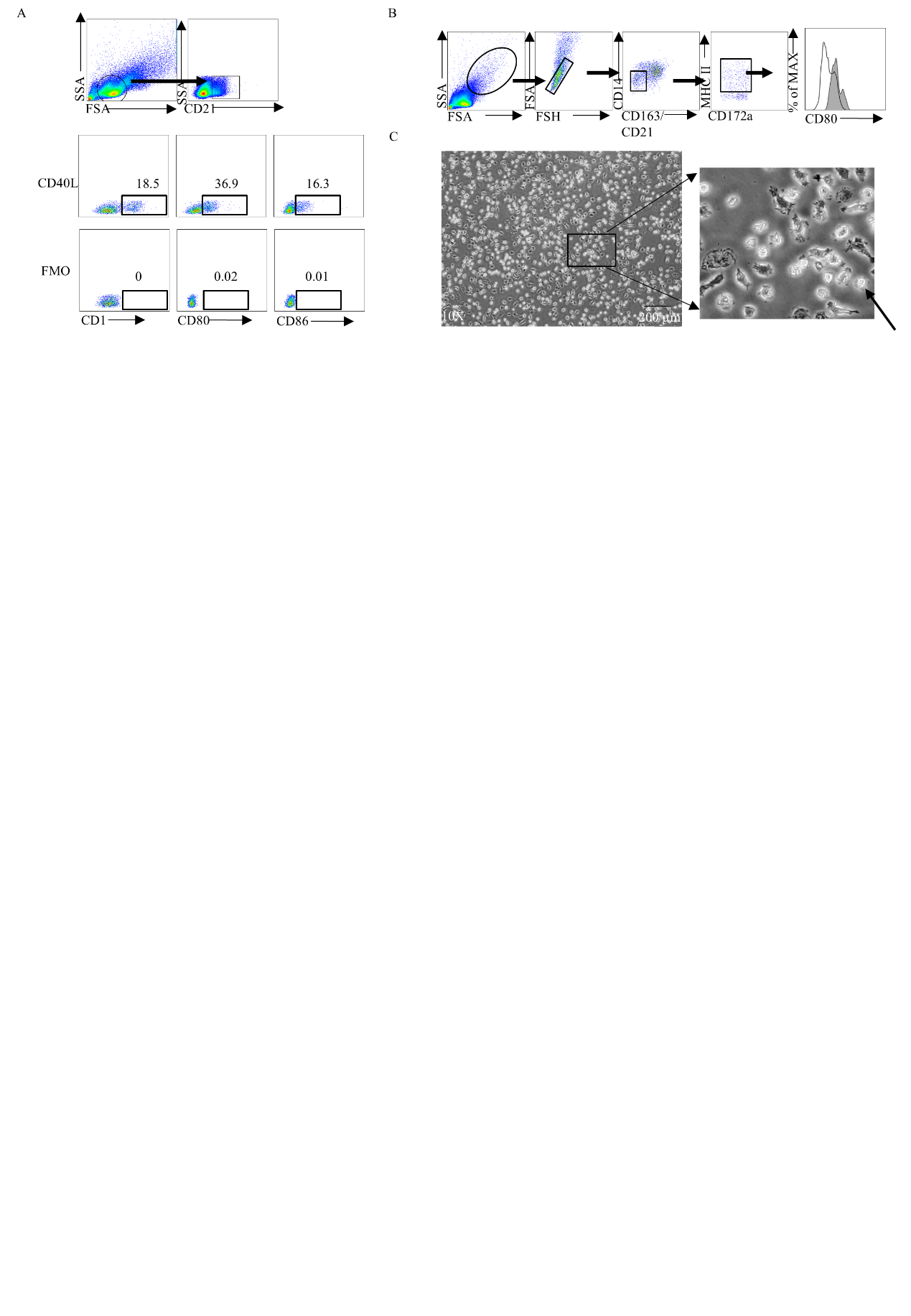
**Supplementary Fig. 2** Analysis of activation of B cells and DCs induced by trimeric CD40L

The gating strategy of B cells. Gated lymphocytes based on FSC-A/SC-A characteristics. (A) Identify B cells through CD21^+^antibody. Representative point plots show the expression of CD1, CD80, and CD86 after CD40L stimulation, and fluorescence subtraction control (FMO) is performed. (B) The gating strategy of DC cells. Gated lymphocytes based on FSC-A/SC-A characteristics. By excluding CD163, CD21, and CD14 positive cells through positivity, further selection was made for CD172a and MHC II positive cells. The representative histogram shows the expression of CD80 in Mo DCs (gray histogram), and fluorescence is subtracted from a control (white histogram). (C) Observe the morphology of dendritic cells at designated time points. The white arrow points to the dendrites.


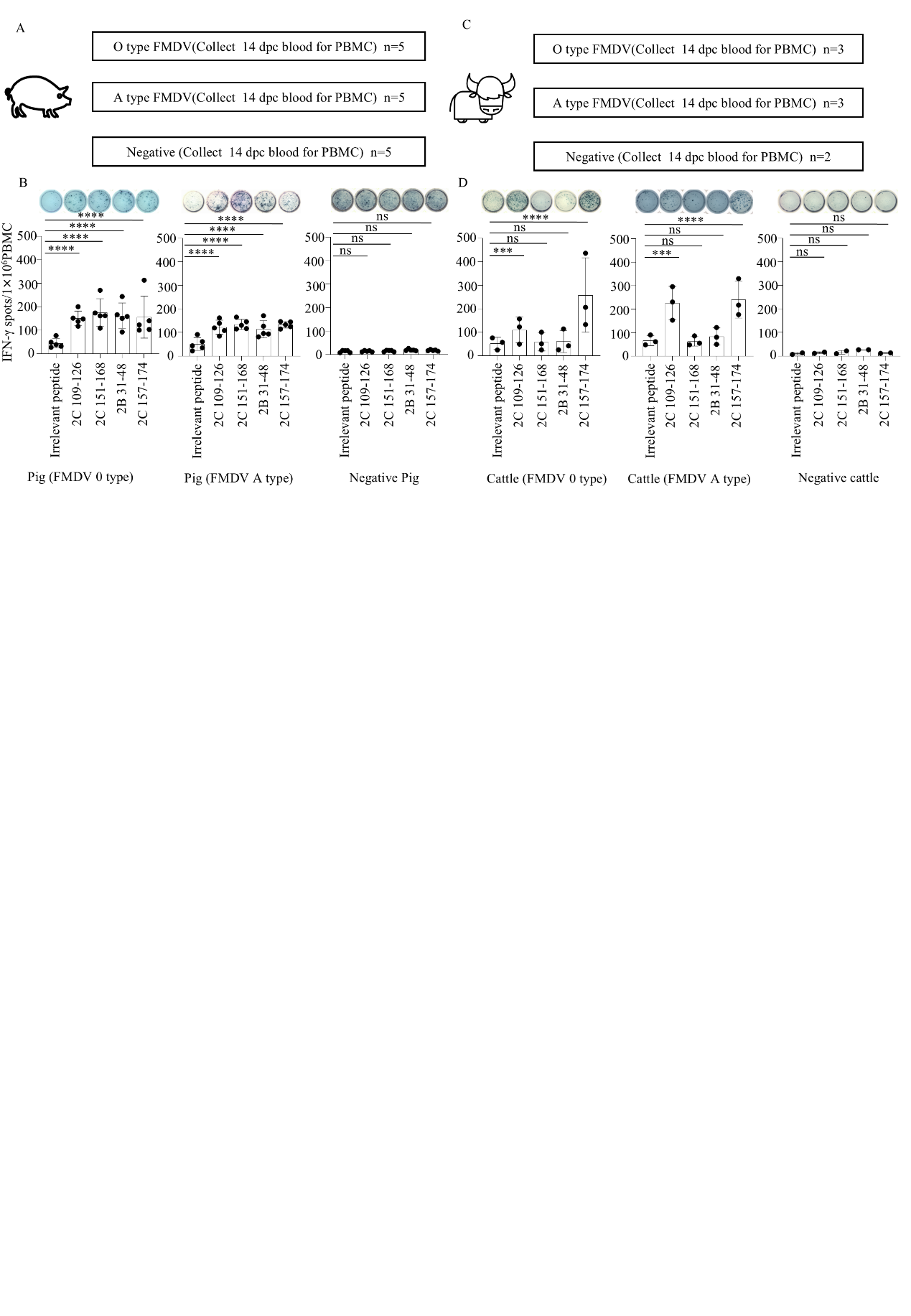
**Supplementary Fig. 3** Broad-spectrum analysis of IFN-γ secretion induced by T cell epitopes in pigs and cattle infected with FMDV serotype O or A. PBMCs were isolated from pigs or cattle infected with FMDV serotype A (n = 5) or O (n = 5) at 14 days post-infection, and the T cell reactivity to 2B and 2C peptides was detected by IFN-γ ELISpot. (A and C) Schematic diagrams of infected animals and sample collection. (B and D) Representative IFN-γ spots of PBMCs stimulated by 2B and 2C peptides, and comparison of the total number of IFN-γ-secreting cells per million PBMCs. Data are presented as mean ± standard deviation (n=5). All data were assessed by one-way ANOVA, ns: no significant difference. **P* < 0.05, ***P* < 0.01, ****P* < 0.001, *****P* < 0.0001.


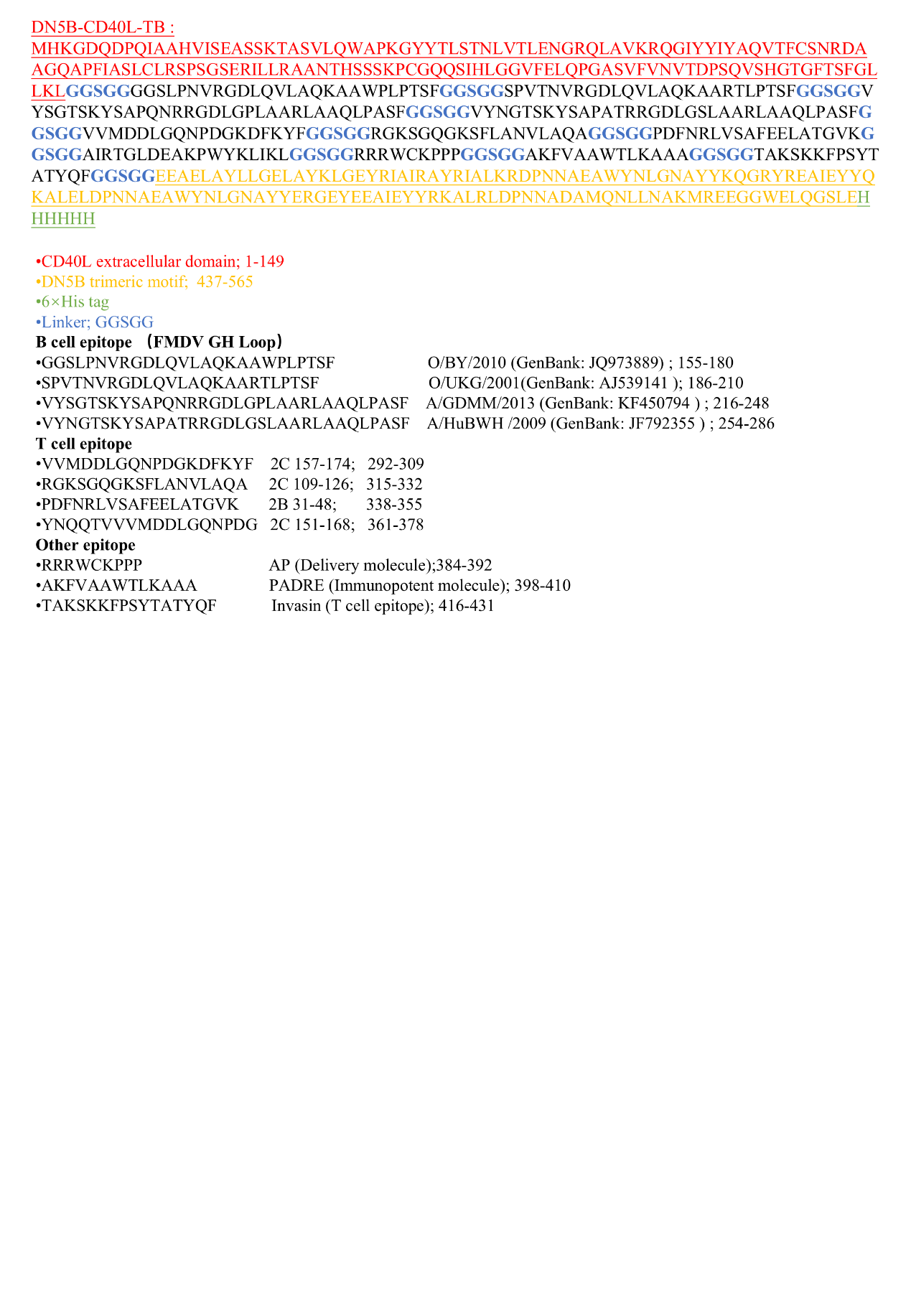


**Supplementary Fig 4.** Trimer-CD40L-TB vaccine sequence information.


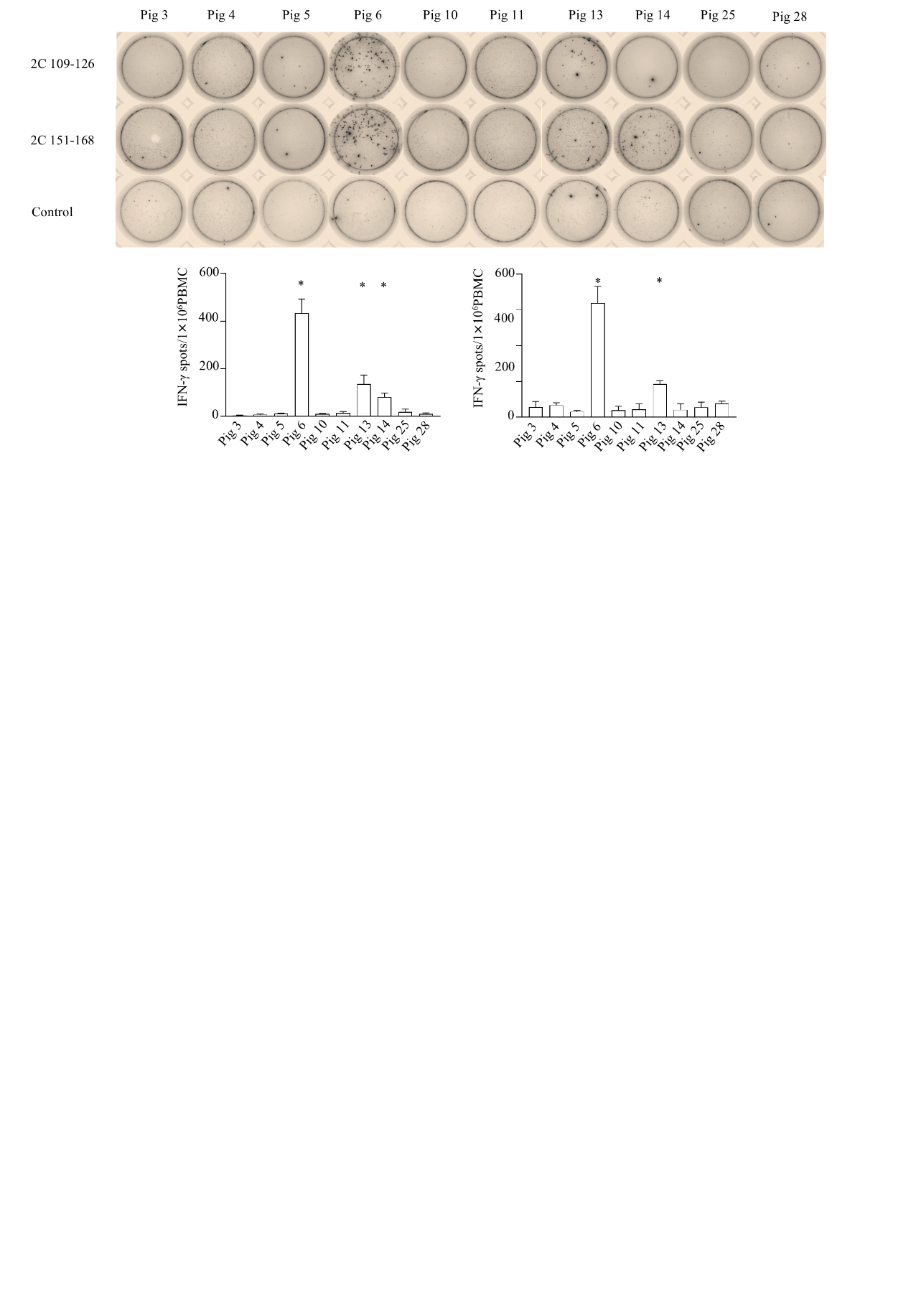
**Supplementary Fig. 5** Analysis of IFN - γ induced by 2C 109-126 and 2C 151-168 under different SLA II backgrounds in pigs.

PBMCs were isolated from Trimer-CD40L-TB (Pig 3-6), Trimer-TB (Pig 10-14) and Trimer (Pigs 25 and 28) group at 35 dpv. 2C 109-126 and 2C 151-168 were used to stimulate PBMCs for 36 h, followed by an ELISpot assay to detect IFN-γ secretion. DMSO served as negative controls. Up figure, representative ELISpot images of reactive individual peptides, negative controls. Down figure data statistics values represent the mean of triplicates of PBMCs from immunized pigs. All data were assessed by one-way ANOVA, ns: no significant difference. **P* < 0.0001.


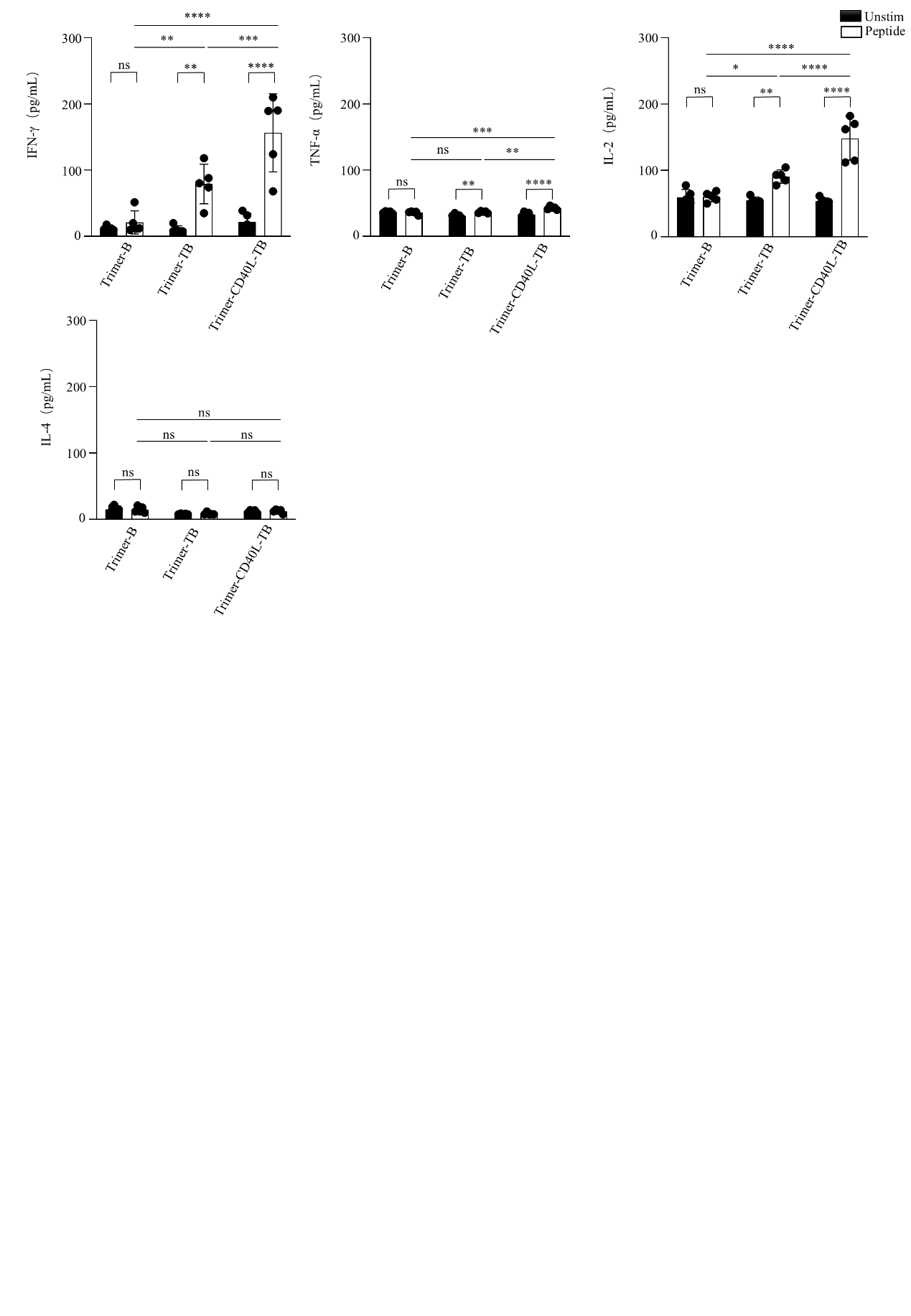


**Supplementary Fig. 6** ELISA analysis of antigen-specific T- cell- mediated immune responses in pig. At 35 dpv, PBMC were collected to determine the cytokine level. PBMC were stimulated with peptide pool (2B 31-48 and 2C 157-174) (n=5/group). The supernatants were tested for cytokine productions by ELISA. All data were assessed by two-way ANOVA, ns: no significant difference. **P* < 0.05, ***P* < 0.01, ****P* < 0.001, *****P* < 0.0001.


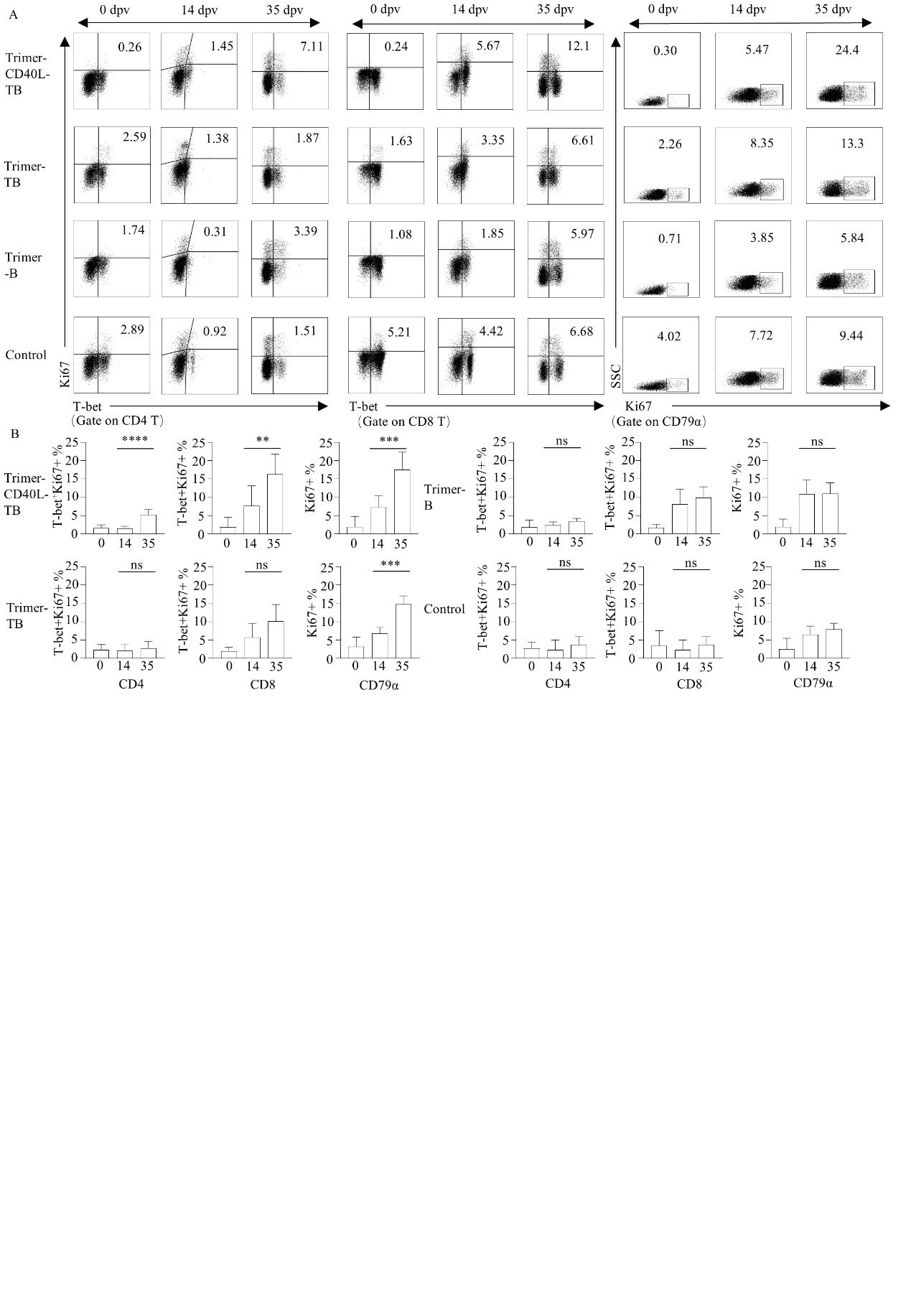


**Supplementary Fig. 7** Analysis of Th1 and CTL nuclear transcription factor T-bet activation and B cell proliferation induced by Trimer-CD40L-TB vaccine immunization. PBMCs were isolated from each group at 0-, 14-, and 35-days post-vaccination (dpv), and the expression of T-bet and Ki67 in CD4⁺ T cells, CD8⁺ T cells, and CD79a⁺ B cells was detected by flow cytometry. (A) Representative dot plots of Ki67 and T-bet expression. (B) Comparison of the percentage differences in T-bet⁺Ki67⁺ and Ki67⁺ expression in CD4⁺ T cells, CD8⁺ T cells, and CD79α⁺ B cells among the four groups. All data were assessed by one-way ANOVA (n=4/group), ns: no significant difference. **P* < 0.05, ***P* < 0.01, ****P* < 0.001, *****P* < 0.0001.

**Supplementary Table 1.** List of overlapping peptides of 2B and 2C from FMDV.

| Name | Sequence | Name | Sequence |
| --- | --- | --- | --- |
| 2B 1-18 | PFFFSDVRSNFSKLVETI | 2C 97-114 | SRSRPEPVVVCLRGKSGQ |
| 2B 7-24 | VRSNFSKLVETINQMQED | 2C 103-120 | PVVVCLRGKSGQGKSFLA |
| 2B 13-30 | KLVETINQMQEDMSTKHG | 2C 109-126 | RGKSGQGKSFLANVLAQA |
| 2B 19-36 | NQMQEDMSTKHGPDFNRL | 2C 115-132 | GKSFLANVLAQAISTHFT |
| 2B 25-42 | MSTKHGPDFNRLVSAFEE | 2C 121-138 | NVLAQAISTHFTGRTDSV |
| 2B 31-48 | PDFNRLVSAFEELATGVK | 2C 127-144 | ISTHFTGRTDSVWYCPPD |
| 2B 37-54 | VSAFEELATGVKAIRTGL | 2C 133-150 | GRTDSVWYCPPDPDHFDG |
| 2B 43-60 | LATGVKAIRTGLDEAKPW | 2C 139-156 | WYCPPDPDHFDGYNQQTV |
| 2B 49-66 | AIRTGLDEAKPWYKLIKL | 2C 145-162 | PDHFDGYNQQTVVVMDDL |
| 2B 55-72 | DEAKPWYKLIKLLSRLSC | 2C 151-168 | YNQQTVVVMDDLGQNPDG |
| 2B 61-78 | YKLIKLLSRLSCMAAVAA | 2C 157-174 | VVMDDLGQNPDGKDFKYF |
| 2B 67-84 | LSRLSCMAAVAARSKDPV | 2C 163-180 | GQNPDGKDFKYFAQMVST |
| 2B 73-90 | MAAVAARSKDPVLVAIML | 2C 169-186 | KDFKYFAQMVSTTGFIPP |
| 2B 79-96 | RSKDPVLVAIMLADTGLE | 2C 175-192 | AQMVSTTGFIPPMASLED |
| 2B 85-102 | LVAIMLADTGLEILDSTF | 2C 181-198 | TGFIPPMASLEDKGKPFN |
| 2B 91-108 | ADTGLEILDSTFVVKKIS | 2C 187-204 | MASLEDKGKPFNSKVIIA |
| 2B 97-114 | ILDSTFVVKKISDSLSSL | 2C 193-210 | KGKPFNSKVIIATTNLYS |
| 2B 103-120 | VVKKISDSLSSLFHVPAP | 2C 199-216 | SKVIIATTNLYSGFTPRT |
| 2B 109-126 | DSLSSLFHVPAPVFSFGA | 2C 205-222 | TTNLYSGFTPRTMVCPDA |
| 2B 115-132 | FHVPAPVFSFGAPILLAG | 2C 211-228 | GFTPRTMVCPDALNRRFH |
| 2B 121-138 | VFSFGAPILLAGLVKVAS | 2C 217-234 | MVCPDALNRRFHFDIDVS |
| 2B 127-144 | PILLAGLVKVASSFFRST | 2C 223-240 | LNRRFHFDIDVSAKDGYK |
| 2B 133-150 | LVKVASSFFRSTPEDLER | 2C 229-246 | FDIDVSAKDGYKINNKLD |
| 2B 139-154 | SFFRSTPEDLERAEKQ | 2C 235-252 | AKDGYKINNKLDIIKALE |
| 2C 1-18 | LKARDINDIFAILKNGEW | 2C 241-258 | INNKLDIIKALEDTHTNP |
| 2C 7-24 | NDIFAILKNGEWLVKLIL | 2C 247-264 | IIKALEDTHTNPVAMFQY |
| 2C 13-30 | LKNGEWLVKLILAIRDWI | 2C 253-270 | DTHTNPVAMFQYDCALLN |
| 2C 19-36 | LVKLILAIRDWIKAWIAS | 2C 259-276 | VAMFQYDCALLNGMAVEM |
| 2C 25-42 | AIRDWIKAWIASEEKFVT | 2C 265-282 | DCALLNGMAVEMKRMQQD |
| 2C 31-48 | KAWIASEEKFVTMTDLVP | 2C 271-288 | GMAVEMKRMQQDMFKPQP |
| 2C 37-54 | EEKFVTMTDLVPGILEKQ | 2C 277-294 | KRMQQDMFKPQPPLQNVY |
| 2C 43-60 | MTDLVPGILEKQRDLNDP | 2C 283-300 | MFKPQPPLQNVYQLVQEV |
| 2C 49-66 | GILEKQRDLNDPSKYKEA | 2C 289-306 | PLQNVYQLVQEVIDRVEL |
| 2C 55-72 | RDLNDPSKYKEAKEWLDN | 2C 295-312 | QLVQEVIDRVELHEKVSS |
| 2C 61-78 | SKYKEAKEWLDNARQACL | 2C 301-318 | IDRVELHEKVSSHPIFKQ |
| 2C 67-84 | KEWLDNARQACLKSGNIH | Irrelevant  SVA* 3A | APDADPVGRLAILAKLGLAL |
| 2C 73-90 | ARQACLKSGNIHIANLCK |  |  |
| 2C 79-96 | KSGNIHIANLCKVVAPAP |  |  |
| 2C 85-102 | IANLCKVVAPAPSRSRPE |  |  |
| 2C 91-108 | VVAPAPSRSRPEPVVVCL |  |  |

Note: *Senecavirus A.

**Supplementary Table 2.** Single peptide composition of 19 peptide libraries

|  | **1** | **2** | **3** | **4** | **5** | **6** | **7** | **8** | **9** | **10** | **11** | **12** |
| --- | --- | --- | --- | --- | --- | --- | --- | --- | --- | --- | --- | --- |
| **13** | 2B 1-18 | 2B 7-24 | 2B 13-30 | 2B 19-36 | 2B 25-42 | 2B 31-48 | 2B 37-54 | 2B 43-60 | 2B 49-66 | 2B 55-72 | 2B 61-78 | 2B 67-84 |
| **14** | 2B 73-90 | 2B 79-96 | 2B 85-102 | 2B 91-108 | 2B 97-114 | 2B 103-120 | 2B 109-126 | 2B 115-132 | 2B 121-138 | 2B 127-144 | 2B 133-150 | 2B 139-154 |
| **15** | 2C 1-18 | 2C 7-24 | 2C 13-30 | 2C 19-36 | 2C 25-42 | 2C 31-48 | 2C 37-54 | 2C 43-60 | 2C 49-66 | 2C 55-72 | 2C 67-84 | 2C 61-78 |
| **16** | 2C 73-90 | 2C 79-96 | 2C 85-102 | 2C 91-108 | 2C 97-114 | 2C 103-120 | 2C 109-126 | 2C 115-132 | 2C 121-138 | 2C 127-144 | 2C 133-150 | 2C 139-156 |
| **17** | 2C 145-162 | 2C 151-168 | 2C 157-174 | 2C 163-180 | 2C 169-186 | 2C 175-192 | 2C 181-198 | 2C 187-204 | 2C 193-210 | 2C 199-216 | 2C 205-222 | 2C 211-228 |
| **18** | 2C 217-234 | 2C 223-240 | 2C 229-246 | 2C 235-252 | 2C 241-258 | 2C 247-264 | 2C 253-270 | 2C 259-276 | 2C 265-282 | 2C 271-288 | 2C 277-294 | 2C 283-300 |
| **19** | 2C 289-306 | 2C 295-312 | 2C 301-318 | DMSO | DMSO | DMSO | DMSO | DMSO | DMSO | DMSO | DMSO | DMSO |

**Supplementary Table 3.** Monoclonal antibodies for FACS analysis.

| **Antigen** | **Clone** | **Isotype** | **Fluorochrome** | **Source of primary Ab** | **Details of secondary Ab** |
| --- | --- | --- | --- | --- | --- |
| **DC and B cell phenotype assay** | | | | | |
| CD172a | BL1H7 | Mouse IgG1 | FITC | Bio-rad |  |
| CD163 | 2A10/11 | Mouse IgG1 | PE | Bio-rad |  |
| CD80 | 16-10A1 | Armenian hamster IgG2 | Perc-cy5.5 | Biolegend |  |
| CD86 | 1B3 | Mouse IgG1 | BV510 | ThermoFisher | Streptavidin-PE |
| CD21 | BB6-11C9.6 | Mouse IgG1 | Biotin | ThermoFisher |  |
| SLA II DQ | K274.3G8 | Mouse IgG1 | Purified | Bio-rad | Lightning-Linked APC，Abcam |
| SLA II DR | 2E9/13 | Mouse IgG2b | Purified | Bio-rad |  |
| CD14 | Mil2 | Mouse IgG2b | Purified | Bio-rad | ReadiLink™ Rapid mFluor™ Violet 450， AAT Bioquest |
| **Memory T cell assay** | | | | | |
| CD3 | BB23-8E6-8C8 | Mouse IgG2a | PerCP-cy5.5 | BD Pharmingen |  |
| CD4 | 74-12-4 | Mouse IgG2b | PE-Cy™7 | BD Pharmingen |  |
| CD8α | 76-2-11 | Mouse IgG2aκ | Biotin | Southernbiotech | BV 421™ |
| TCRγδ | MAC320 | Rat IgG2a | PE | BD Pharmingen |  |
| CCR7 | 3D12 | Rat IgG2a | BV510 | BD Pharmingen |  |
| CD27 | B30C7 | Mouse IgG1 | APC | Bio-rad |  |
| CD45RA | MIL13 | Mouse IgG1 | FITC | Bio-rad |  |
| **Live/dead cell and Streptavidin antibody** | | | | | |
| Streptavidin |  |  | BV 510™ | Biolegend |  |
| Streptavidin |  |  | BV 421™ | Biolegend |  |
| Fixable Viability Dye eFluor™ 780 |  |  | eFluor™ 780 | eBioscience™ |  |
| **ICS assay** | | |  |  |  |
| CD3 | BB23-8E6-8C8 | Mouse IgG2a | PerCP-cy5.5 | BD Pharmingen |  |
| CD4 | 74-12-4 | Mouse IgG2b | PE-Cy™7 | BD Pharmingen |  |
| CD8α | 76-2-11 | Mouse IgG2aκ | Biotin | Southernbiotech | BV 510™ |
| TCRγδ | MAC320 | Rat IgG2a | PE | BD Pharmingen |  |
| IFN-γ | P2G10 | Mouse IgG1 | APC | BD Pharmingen |  |
| **CD4^+^T-bet^+^, CD8^+^T-bet^+^ and CD79α^+^ proliferation assay** | | | | | |
| CD3 | BB23-8E6-8C8 | Mouse IgG2a | PerCP-cy5.5 | BD Pharmingen |  |
| CD4 | 74-12-4 | Mouse IgG2b | PE-Cy™7 | BD Pharmingen |  |
| CD8α | 76-2-11 | Mouse IgG2aκ | Biotin | Southernbiotech | BV 510™ |
| TCRγδ | MAC320 | Rat IgG2a | PE | BD Pharmingen |  |
| Ki67 | B56 | Mouse IgG1 | BV 421™ | BD Pharmingen |  |
| T-bet | 4B10 | Mouse IgG1 | APC | eBioscience^TM^ |  |
| CD79α | HM47 | Mouse IgG1 | FITC | eBioscience^TM^ |  |
| **CFSE proliferation assay** | | | | | |
| CD3 | BB23-8E6-8C8 | Mouse IgG2a | PerCP-cy5.5 | BD Pharmingen |  |
| CD4 | 74-12-4 | Mouse IgG2b | PE-Cy™7 | BD Pharmingen |  |
| CD8α | 76-2-11 | Mouse IgG2aκ | Biotin | Southernbiotech | BV 510™ |
| TCRγδ | MAC320 | Rat IgG2a | PE | BD Pharmingen |  |
| CFSE |  |  |  | BD Pharmingen |  |
| **Magnetic bead sorting** | | | | | |
| Anti-FITC MicroBeads |  |  |  | BD Pharmingen |  |
| CD8β | PTT3A | Mouse IgG1 | FITC | Thermo |  |
| **SLA blocking antibody** | | | | | |
| SLA I | 74-11-10 | Mouse IgG2b |  | Kingfishers |  |
| SLA II | MSA-3 | Mouse IgG2a |  | Kingfishers |  |
| **GC B cell** | | | | | |
| BCL-6 | K112-91 | Mouse IgG1 | BV 421 | BD Pharmingen |  |
| CD79α | HM47 | Mouse IgG1 | FITC | eBioscience^TM^ |  |
| GH-loop |  |  | Biotin |  | APC |

**Supplementary Table 4.** Possible identified from peptide library 24 IFN-γ reactive single peptides.

| **1** | **2** | **3** | **4** | **5** | **6** | **7** | **8** | **9** | **10** | **11** | **12** |
| --- | --- | --- | --- | --- | --- | --- | --- | --- | --- | --- | --- |
| 2B 7-24 | 2B 13-30 | 2B 25-42 | 2B 31-48 | 2B 37-54 | 2B 49-66 | 2B 79-96 | 2B 85-102 | 2B 97-114 | 2B 103-120 | 2B 109-126 | 2B 121-138 |
| **13** | **14** | **15** | **16** | **17** | **18** | **19** | **20** | **21** | **22** | **23** | **24** |
| 2C 79-96 | 2C 85-102 | 2C 97-114 | 2C 103-120 | 2C 109-126 | 2C 122-138 | 2C 151-168 | 2C 157-174 | 2C 169-186 | 2C 175-192 | 2C 181-198 | 2C 193-210 |

**Supplementary Table 5.** Genbank information for 7 serotypes of FMDV

| Type A | Type O | Type C | Asia I | SAT I | SAT II | SAT III |
| --- | --- | --- | --- | --- | --- | --- |
| MT863268 | GU384682 | FJ824812 | DQ533483 | MN275121 | MH053337 | MH053341 |
| MT495472 | MN953620 | AY593805 | EF149010 | MH053324 | MT602089 | MW355679 |
| KT968663 | AF506822 | KY825722 | EF614458 | AY593842 | MW715627 | AY593850 |
| KY322678 | AJ539141 | MH053310 | HQ63136 | MT602085 | KU821592 | MG372727 |
| HQ632773 | HM008917 | MH053308 |  | MT602088 |  | MH053350 |
| KY322677 | HQ009509 | MH05330 |  |  |  |  |
| OK318501 | KY444645 |  |  |  |  |  |
| OM455473 | JN998085 |  |  |  |  |  |
| MH053305 | JQ973889 |  |  |  |  |  |
| AY593764 | HQ412603 |  |  |  |  |  |
| AY593765 | KU204893 |  |  |  |  |  |

|  | SLA II DRA | SLA II DRB | SLA II DQA | SLA II DQB |
| --- | --- | --- | --- | --- |
| **Screening T-cell epitopes** | | | | |
| Pig 55 | 02:02:02 | 10:01 | 01:01 | 06:01 |
| Pig 67 | 02:02:02 | 04:05 | 01:04 | 06:03 |
| Pig 68 | 01:01:04 | 09:06 | 02:05 | 02:01 |
| **Trimer-CD40L-TB group** | | | | |
| Pig 1 | 02:02:01 | 06:07 | 01:06 | 01:01 |
| Pig 2 | 01:01:04 | 06:07 | 01:01:02 | 01:01 |
| Pig 3 | 01:01:03 | 01:01 | 01:03 | 06:01 |
| Pig 4 | 01:01:04 | 01:01 | 01:01 | 07:01:02 |
| Pig 5 | 02:02:03 | 06:07 | 01:03 | 01:01 |
| Pig 6 | 02:02:01 | 10:01 | 01:01 | 06:03 |
| **Trimer-TB group** | | | | |
| Pig 9 | 02:02:01 | 01:01 | 01:01 | 06:01 |
| Pig 10 | 01:01:03 | 06:01 | 01:01 | 01:01 |
| Pig 11 | 02:02:02 | 06:07 | 01:03 | 07:01 |
| Pig 12 | 02:02:03 | 06:07 | 01:02 | 07:01 |
| Pig 13 | 02:02:01 | 10:01 | 01:02 | 07:01 |
| Pig 14 | 01:01:03 | 01:01 | 01:01 | 06:03 |

**Supplementary Table 6. SLA II type variance analysis**
